# Supra­molecular hy­dro­gen-bonded networks formed from copper(II) car­box­yl­ate dimers

**DOI:** 10.1107/S2053229624004534

**Published:** 2024-05-22

**Authors:** Brendan F. Abrahams, Christopher J. Commons, Timothy A. Hudson, Robin Sanchez-Arlt

**Affiliations:** a University of Melbourne, School of Chemistry, Grattan St, Parkville, 3052, Australia; University of Monash, Australia

**Keywords:** crystal structure, hy­dro­gen-bonded network, 4-hy­droxy­benzoic acid, short hy­dro­gen bond, crystal engineering, topology, paddle wheel complex, car­box­yl­ate dimer

## Abstract

The well-known copper car­box­yl­ate dimer, with four car­box­yl­ate ligands ex­ten­ding outwards towards the corners of a square, has been employed to generate a series of crystalline com­pounds. In particular, this work centres on the use of the 4-hy­droxy­benzoate anion (Hhba^−^) and its deprotonated phe­nol­ate form 4-oxidobenzoate (hba^2−^) to obtain complexes with the general formula [Cu_2_(Hhba)_4–*x*_(hba)*_x_L*_2–*y*_]^*x*−^, where *L* is an axial coligand (including solvent mol­ecules), *x* = 0, 1 or 2, and *y* = 0 or 1. In some cases, short hy­dro­gen bonds result in complexes which may be represented as [Cu_2_(Hhba)_2_(H_0.5_hba)_2_ *L*_2_]^−^. The main focus of the investigation is on the formation of a variety of extended networks through hy­dro­gen bonding and, in some crystals, coordinate bonds when bridging coligands (*L*) are employed. Crystals of [Cu_2_(Hhba)_4_(di­ox­ane)_2_]·4(di­ox­ane) consist of the expected Cu dimer with the Hhba^−^ anions forming hy­dro­gen bonds to 1,4-di­ox­ane mol­ecules which block network formation. In the case of crystals of com­position [Et_4_N][Cu_2_(Hhba)_2_(H_0.5_hba)_2_(CH_3_OH)(H_2_O)]·2(di­ox­ane), Li[Cu_2_(Hhba)_2_(H_0.5_hba)_2_(H_2_O)_2_]·3(di­ox­ane)·4H_2_O and [Cu_2_(Hhba)_2_(H_0.5_hba)_2_(H_0.5_DABCO)_2_]·3CH_3_OH (DABCO is 1,4-di­aza­bicyclo­[2.2.2]octa­ne), square-grid hy­dro­gen-bonded networks are generated in which the complex serves as one type of 4-con­necting node, whilst a second 4-con­necting node is a hy­dro­gen-bonding motif assembled from four phenol/phenolate groups. Another two-dimensional (2D) network based upon a related square-grid structure is formed in the case of [Et_4_N]_2_[Cu_2_(Hhba)_2_(hba)_2_(di­ox­ane)_2_][Cu_2_(Hhba)_4_(di­ox­ane)(H_2_O)]·CH_3_OH. In [Cu_2_(Hhba)_4_(H_2_O)_2_]·2(Et_4_NNO_3_), a square-grid structure is again apparent, but, in this case, a pair of nitrate anions, along with four phenolic groups and a pair of water mol­ecules, combine to form a second type of 4-con­necting node. When 1,8-bis­(di­methyl­amino)­naphthalene (bdn, ‘proton sponge’) is used as a base, another square-grid network is generated, *i.e.* [Hbdn]_2_[Cu_2_(Hhba)_2_(hba)_2_(H_2_O)_2_]·3(di­ox­ane)·H_2_O, but with only the copper dimer complex serving as a 4-con­necting node. Complex three-dimensional networks are formed in [Cu_2_(Hhba)_4_(O-bipy)]·H_2_O and [Cu_2_(Hhba)_4_(O-bipy)_2_]·2(di­ox­ane), where the potentially bridging 4,4′-bi­pyridine *N*,*N*′-dioxide (O-bipy) ligand is employed. Rare cases of mixed car­box­yl­ate copper dimer complexes were obtained in the cases of [Cu_2_(Hhba)_3_(OAc)(di­ox­ane)]·3.5(di­ox­ane) and [Cu_2_(Hhba)_2_(OAc)_2_(DABCO)_2_]·10(di­ox­ane), with each structure possessing a 2D network structure. The final com­pound re­por­ted is a simple hy­dro­gen-bonded chain of com­position (H_0.5_DABCO)(H_1.5_hba), formed from the reaction of H_2_hba and DABCO.

## Abstract 2

Outward-directing phenolic/phenolate groups on copper car­box­yl­ate dimers have a powerful structure-directing influence, leading to the generation of an assortment of hy­dro­gen-bonded networks.

## Introduction

In 1953, the structure of the copper(II) acetate tetra-μ-acetato-bis­[aqua­copper(II)], [Cu_2_(OAc)_4_(H_2_O)_2_], was re­por­ted and shown to consist of a pair of Cu^II^ centres bridged by four car­box­yl­ate groups (van Niekerk & Schoening, 1953 ▸). This remarkable com­pound, possessing a beautiful blue colour and an elegant structure, is one of the classic coordination complexes and, as a result, is a common synthetic target in undergraduate chemistry laboratory programs. The four car­box­yl­ate ligands extend outwards towards the vertices of an approximate square, and thus the binuclear unit has been employed as a square-planar con­nector in both discrete and polymeric supra­molecular systems formed from bridging ligands possessing two or more car­box­yl­ate groups (Eddaoudi *et al.*, 2001 ▸; Chui *et al.*, 1999 ▸; Lee *et al.*, 2021 ▸). The binuclear tetra­car­box­yl­ate moiety has been commonly referred to as a ‘paddle wheel unit’, with the Cu⋯Cu direction colinear with the ‘wheel axle’ and the car­box­yl­ate groups serving as ‘paddles’.

Over the last decade our group has been inter­ested in coordination polymers formed from the combination of metal ions with the anions of 4-hy­droxy­benzoic acid (H_2_hba; Scheme 1), resulting in a variety of polymeric networks with com­positions such as Zn(hba) and Co(hba) (White *et al.*, 2015 ▸), and Cu_3_(hba)_2_(OH)_2_ (Abrahams *et al.*, 2022 ▸), which formed as solvates. Whilst these networks contain the hba^2−^ ligand in the presence of transition-metal ions, the reactions with Group 1 and 2 metal ions often failed to remove the phenolic proton of H_2_hba, even when the metal hydroxide was employed. These reactions commonly resulted in only the Hhba^−^ monoanion being formed (Abrahams *et al.*, 2021 ▸). In some instances, the ligand formed a strong hy­dro­gen bond with an adjacent ligand to create a dimeric unit with a 1− charge, *i.e.* (H_1.5_hba)_2_. In this unit, the O atoms involved are closely separated (approximately 2.45 Å), with the H atom between the two halves of the dimer either at the mid-point of the two O atoms or disordered over two closely separated positions. 
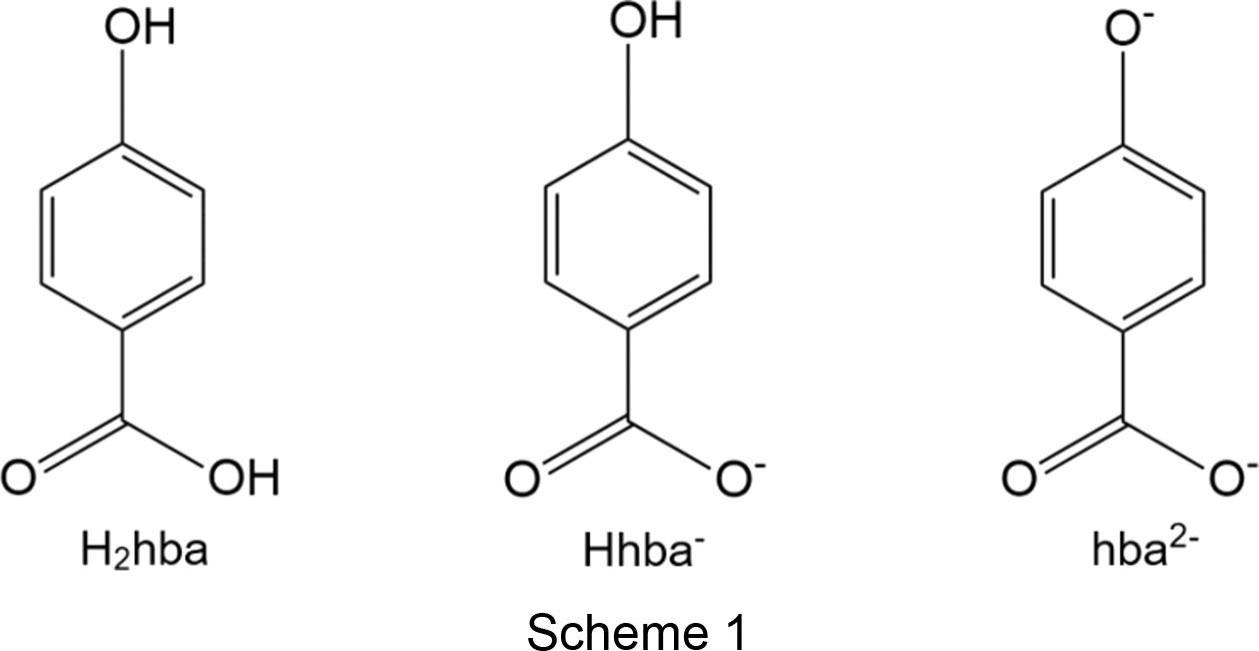


The observation that the phenolic protons were retained in the generation of hy­dro­gen-bonded networks prompted an investigation into whether the binuclear Cu^II^ dimer unit, combined with 4-hy­droxy­benzoic acid in either its monoanionic or dianionic form, was able to serve as a square-planar hy­dro­gen-bonding unit in extended structures. Within such structures, negatively charged phenolate groups could serve as hy­dro­gen-bond acceptors, whilst phenolic groups could serve as both acceptors and donors. This current work describes a series of 11 com­pounds (**1**–**11**) involving Hhba^−^/hba^2−^ ligands in which the geometry of the Cu dimer leads to the generation of a variety of hy­dro­gen-bonded complexes and networks. The structure of a 1:1 cocrystal (**12**) formed from the combination of H_2_hba and DABCO (DABCO is 1,4-di­aza­bicyclo­[2.2.2]octa­ne) is also re­por­ted. Compound **12** serves as a convenient starting material for the generation of com­pound **4**.

## Experimental

### Synthesis and crystallization

**1**–**11** are com­pounds of copper. Compound **12** is a cocrystal of H_2_hba and DABCO, denoted (H_0.5_DABCO)(H_1.5_hba), which was used in the synthesis of **4**.

#### [Cu_2_(Hhba)_4_(di­ox­ane)_2_]·4(di­ox­ane), 1

Cu(OAc)_2_·H_2_O (0.40 g, 2.0 mmol) and H_2_hba (0.55 g, 4.0 mmol) were dissolved in 1,4-di­ox­ane (40 ml). The solution was heated, with stirring, for 2.5 h at approximately 80 °C. The solvent was allowed to evaporate slowly and blue crystals separated from the solution after several days.

#### [NEt_4_][Cu_2_(Hhba)_2_(H_0.5_hba)_2_(CH_3_OH)(H_2_O)]·2(di­ox­ane), 2

Cu(OAc)_2_·H_2_O (0.10 g, 0.50 mmol) and H_2_hba (0.28 g, 2.0 mmol) were dissolved in 1,4-di­ox­ane (10 ml). The solution was heated, with stirring, for 1.5 h at approximately 80 °C. Methanol (10 ml) and water (1 ml) were added, fol­lowed by the dropwise addition, with stirring, of a 25 wt% solution of Et_4_NOH in water (1.3 ml). The solvent was allowed to evaporate slowly and blue crystals separated from solution after several days.

#### Li[Cu_2_(Hhba)_2_(H_0.5_hba)_2_(H_2_O)_2_]·3(di­ox­ane)·4H_2_O, 3

Cu(OAc)_2_·H_2_O (0.40 g, 2.0 mmol) and H_2_hba (0.55 g, 4.0 mmol) were dissolved in 1,4-di­ox­ane (30 ml). The solution was heated, with stirring, for 2.5 h at approximately 80 °C.

To a 2 ml sample of this solution was added LiOH·H_2_O (0.011 g, 0.27 mmol) and me­tha­nol (1 ml). The solvent was allowed to evaporate slowly and a mixture of dark-green crystals of **3** and pale-green crystals separated from the solution after several days. The pale-green crystals were shown by X-ray diffraction to be the mononuclear mol­ecular complex [Cu(Hhba)_2_(H_2_O)_3_]·H_2_O. The structure of this complex has been re­­por­ted previously [Cambridge Structural Database (CSD) refcodes BAPHEF (Shnulin *et al.*, 1981 ▸) and ICUJUN (Liu & Li, 2007 ▸); CSD Version 5.45, March 2024 release; Groom *et al.*, 2016 ▸].

#### [Cu_2_(Hhba)_2_(H_0.5_hba)_2_(H_0.5_DABCO)_2_]·3CH_3_OH, 4

Cu(OAc)_2_·H_2_O (0.016 g, 0.080 mmol) and (H_0.5_DABCO)(H_1.5_hba) (0.46 g, 1.3 mmol) were dissolved in CH_3_OH (12 ml). The solvent was allowed to evaporate slowly and dark-green crystals formed after one day. [The synthesis of (H_0.5_DABCO)(H_1.5_hba), **12**, is described below.]

#### [Et_4_N]_2_[Cu_2_(Hhba)_2_(hba)_2_(di­ox­ane)_2_][Cu_2_(Hhba)_4_(di­ox­ane)(H_2_O)]·CH_3_OH, 5

Cu(OAc)_2_·H_2_O (0.040 g, 0.20 mmol) and H_2_hba (0.055 g, 0.040 mmol) were dissolved in 1,4-di­ox­ane (10 ml). The solution was heated, with stirring, for 4 h at approximately 80 °C and me­tha­nol (10 ml) was added. A few drops of a 25 wt% solution of Et_4_NOH in water were added slowly with stirring until the precipitate that formed upon addition of each drop only just dissolved. Solvent was allowed to evaporate slowly and dark-blue crystals separated from solution after several days.

#### [Cu_2_(Hhba)_4_(H_2_O)_2_]·2Et_4_N(NO_3_), 6

H_2_hba (0.55 g, 4.0 mmol) was added to a 25 wt% solution of Et_4_NOH in water (8.0 ml) and heated, with stirring, at approximately 80 °C for 30 min. Water was evaporated from the solution and the resulting white solid dried by vacuum sublimation. A solution of the white solid (0.10 g) in me­tha­nol (2 ml) was added to a solution of Cu(NO_3_)_2_·3H_2_O (0.10 g, 0.41 mmol) in me­tha­nol (2 ml). A yellow–green precipitate formed immediately which redissolved with stirring. The solvent was allowed to evaporate slowly and blue crystals separated from the solution after several days.

#### [Hbdn]_2_[Cu_2_(Hhba)_2_(hba)_2_(H_2_O)_2_]·3(di­ox­ane)·H_2_O, 7

Cu(OAc)_2_·H_2_O (0.10 g, 0.50 mmol) and H_2_hba (0.14 g, 1.0 mmol) were added to 1,4-di­ox­ane (10 ml). The solution was heated, with stirring, at approximately 70 °C for 10 min. 1,8-Bis(di­methyl­amino)­naphthalene (bdn, ‘proton sponge’; 0.070 g, 0.33 mmol) and aceto­nitrile (2 ml) were added, and the mixture heated for a further 30 min. The solvent was allowed to evaporate slowly and green crystals separated from solution after several days.

#### [Cu_2_(Hhba)_4_(O-bipy)]·H_2_O, 8

Cu(OAc)_2_·H_2_O (0.20 g, 1.0 mmol), 4,4′-bi­pyridine *N*,*N*′-dioxide (O-bipy; 0.10 g, 0.53 mmol) and H_2_hba (0.20 g, 1.4 mmol) were added to water (10 ml) and xylene (1 ml). The mixture was heated at 110 °C in a Teflon-lined autoclave for 5 h to yield dark-green crystals.

#### [Cu_2_(Hhba)_4_(O-bipy)_2_]·2(di­ox­ane), 9

Cu(OAc)_2_·H_2_O (0.10 g, 0.50 mmol), O-bipy (0.050 g, 0.27 mmol) and H_2_hba (0.050 g, 0.36 mmol) were added to 1,4-di­ox­ane (10 ml). The mixture was heated at 110 °C in a Teflon-lined auto­clave for 1 d to yield dark-green crystals.

#### [Cu_2_(Hhba)_3_(OAc)(di­ox­ane)]·3.5di­ox­ane, 10

Cu(OAc)_2_·H_2_O (0.40 g, 2.0 mmol) and H_2_hba (0.55 g, 4.0 mmol) were dissolved in 1,4-di­ox­ane (40 ml). The solution was heated, with stirring, for 4 h at approximately 80 °C. The sol­vent was allowed to evaporate slowly and dark-blue crystals separated from solution after several days.

#### <!?up>[Cu_2_(Hhba)_2_(OAc)_2_(DABCO)_2_]·10(di­ox­ane), 11

Cu(OAc)_2_·H_2_O (0.10 g, 0.50 mmol), H_2_hba (0.069 g, 0.50 mmol) and 1,4-di­aza­bicyclo­[2.2.2]octane (DABCO; 0.061 g, 0.54 mmol) were added to 1,4-di­ox­ane (50 ml). A 0.01 *M* NaOH solution (50 µl) was then added. The mixture was heated at 115 °C in a Teflon-lined autoclave for 2 d to yield small green crystals.

#### (H_0.5_DABCO)(H_1.5_hba), 12

Separate 5 ml me­tha­nol solutions of H_2_hba (1.34 g, 10 mmol) and DABCO (2.75 g, 25 mmol) were prepared. The two solutions were heated to approximately 40 °C, mixed and then heated for a further 15 min. Acetone (20 ml) was added and the solution allowed to evaporate. Colourless crystals formed after approximately 1 h.

### Refinement

Crystal data, data collection and structure refinement details are summarized in Table 1 ▸. The H atoms of water mol­ecules, phenolic groups and carb­oxy­lic acid groups were commonly located in difference Fourier maps and were generally refined with O—H distances restrained to 0.85 Å and *U*_iso_(H) values of 1.5*U*_eq_(O).

In **2**, H atoms were involved in short hy­dro­gen bonds (O⋯O distances less than 2.45 Å) between the symmetry-related O atoms of phenolate groups, and single peaks were observed in difference Fourier maps midway between the atoms. These peaks were assigned as H atoms and refined independently. Short hy­dro­gen bonds were also present in **3** and **4** between the symmetry-related O atoms of phenol groups; however, single peaks midway between the O atoms were not observed. In these cases, H atoms were located on both O atoms with 0.5 occupancies and O—H distances restrained to 0.85 Å.

The H_2_hba and DABCO mol­ecules are closely aligned in **12** (N⋯O distances of approximately 2.52 Å) and single peaks were observed in difference Fourier maps almost midway between the N and O atoms. These peaks were assigned as H atoms and refined independently.

Other H atoms were placed in calculated positions and refined as riding atoms, with *U*_iso_(H) = 1.2*U*_eq_(C) for methyl­ene and aromatic C atoms, *U*_iso_(H) = 1.5*U*_eq_(C) for methyl C atoms, and with C—H = 0.95 Å for aromatic groups, 0.99 Å for methyl­ene group and 0.98 Å for methyl groups. The H atoms of two disordered water mol­ecules (containing O11 and O13) were not modelled in **3**. Details of the refinements can be found in the embedded CIF files.

The mask process within *OLEX2* (Dolomanov *et al.*, 2009 ▸) was employed in the structure refinements for **4**, **7** and **10**, which represent structures for which not all solvent mol­ecules could be satisfactorily modelled.

## Results and discussion

**Figure 1:**
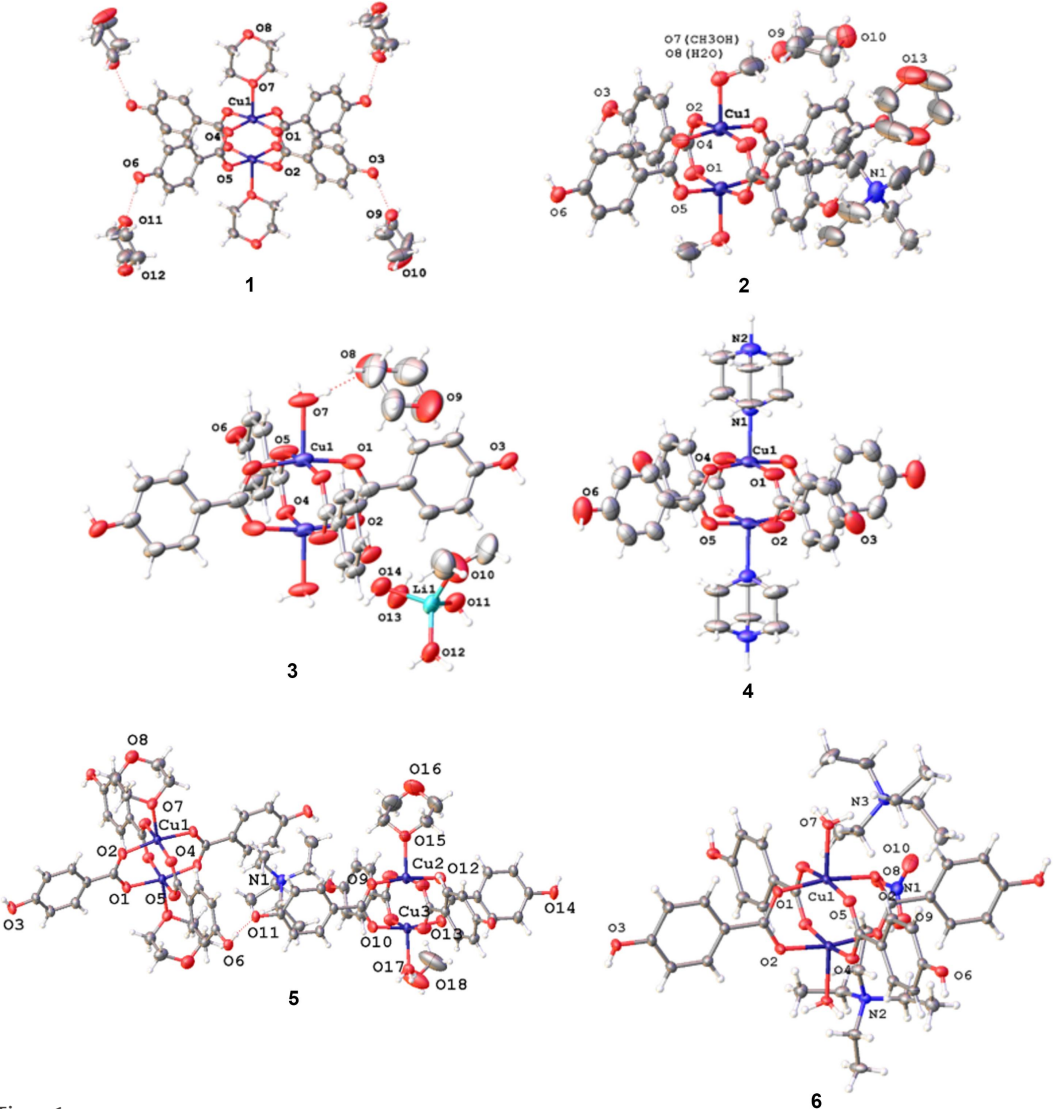
The structures of com­pounds **1**–**6**, showing the copper dimer units and the atom-labelling schemes. For clarity, the labels of the C and H atoms are not shown. Displacement ellipsoids are drawn at the 50% probability level. H atoms are represented by spheres of arbitrary size. The red dotted lines represent hy­dro­gen-bonding inter­actions. In **2**, only one configuration of the disordered di­ox­ane mol­ecules is shown, and in **4**, only one configuration of the DABCO mol­ecules is shown.

Labelled structures of com­pounds **1**–**12** showing displacement ellipsoids for non-H atoms are presented in Figs. 1 ▸ and 2 ▸.

**Figure 3:**
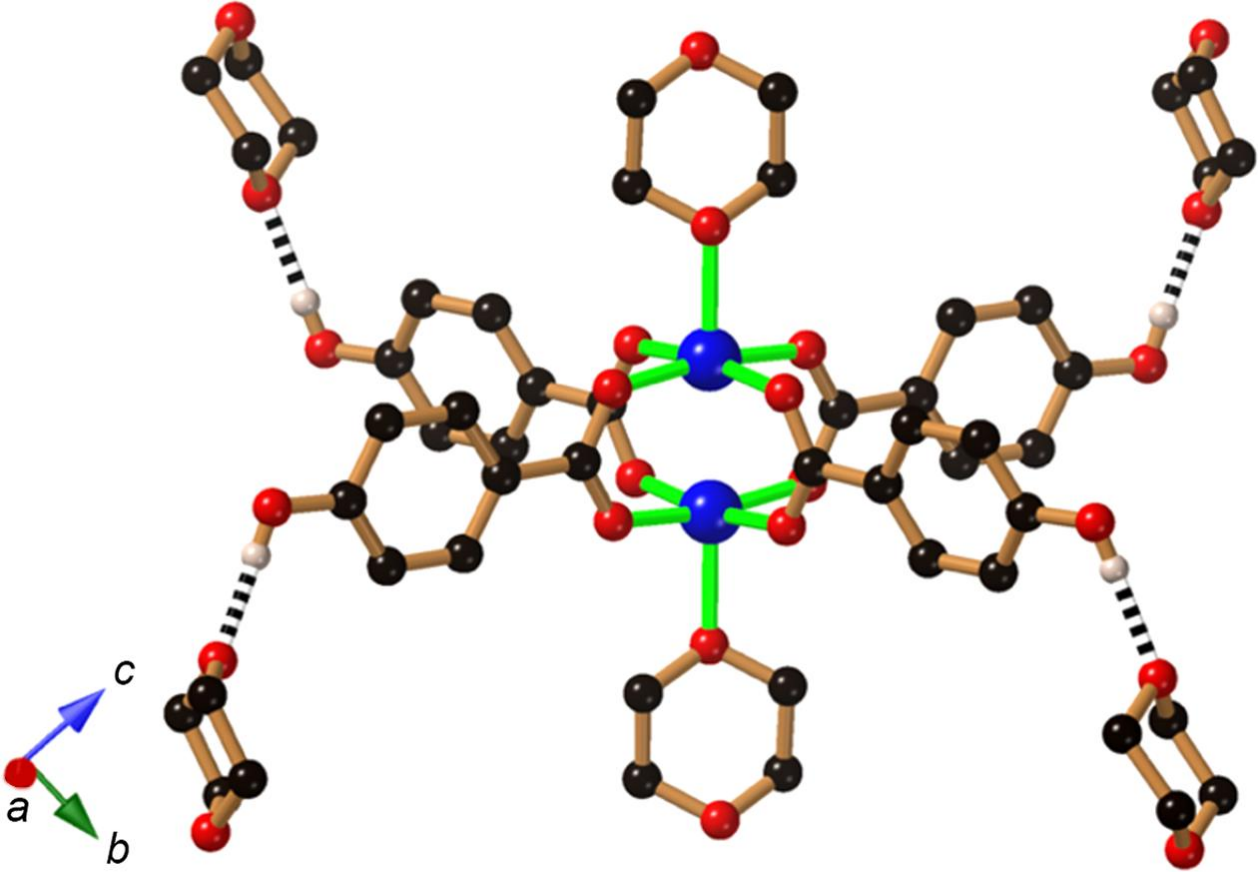
The structure of [Cu_2_(Hhba)_4_(di­ox­ane)_2_]·4(di­ox­ane) (**1**), showing the links to two coordinated di­ox­ane mol­ecules and hy­dro­gen-bond inter­actions with four non-coordinated di­ox­ane mol­ecules. Colour code for this and later figures, as applicable: Cu dark blue, C black, O red, N and Li pale blue, and H pale pink. Hydrogen bonds are indicated by black and white striped con­nections. For clarity, H atoms not involved in hy­dro­gen bonding have been omitted in this and most of the following figures.

Our general strategy towards the synthesis of hy­dro­gen-bonded copper(II) complexes incorporating Hhba^−^ anions involves the addition of H_2_hba to Cu_2_(OAc)_4_·2H_2_O. In this reaction, the acetate anion can serve as the base for H_2_hba. The combination of Cu_2_(OAc)_4_·2H_2_O with H_2_hba in a 1:4 ratio in 1,4-di­ox­ane (di­ox­ane) yields a com­pound of com­position [Cu_2_(Hhba)_4_(di­ox­ane)_2_]·4(di­ox­ane) (**1**). As anti­ci­pated, the dimeric copper car­box­yl­ate complex is formed, as indicated in Fig. 3 ▸, with the Hhba^−^ units ex­ten­ding towards the vertices of an approximate square. Each phenolic group of the coordinated Hhba^−^ serves as a proton donor to a di­ox­ane mol­ecule. In addition, a di­ox­ane mol­ecule is coordinated to opposing axial sites on each of the symmetry-related Cu^II^ centres.

**Figure 4:**
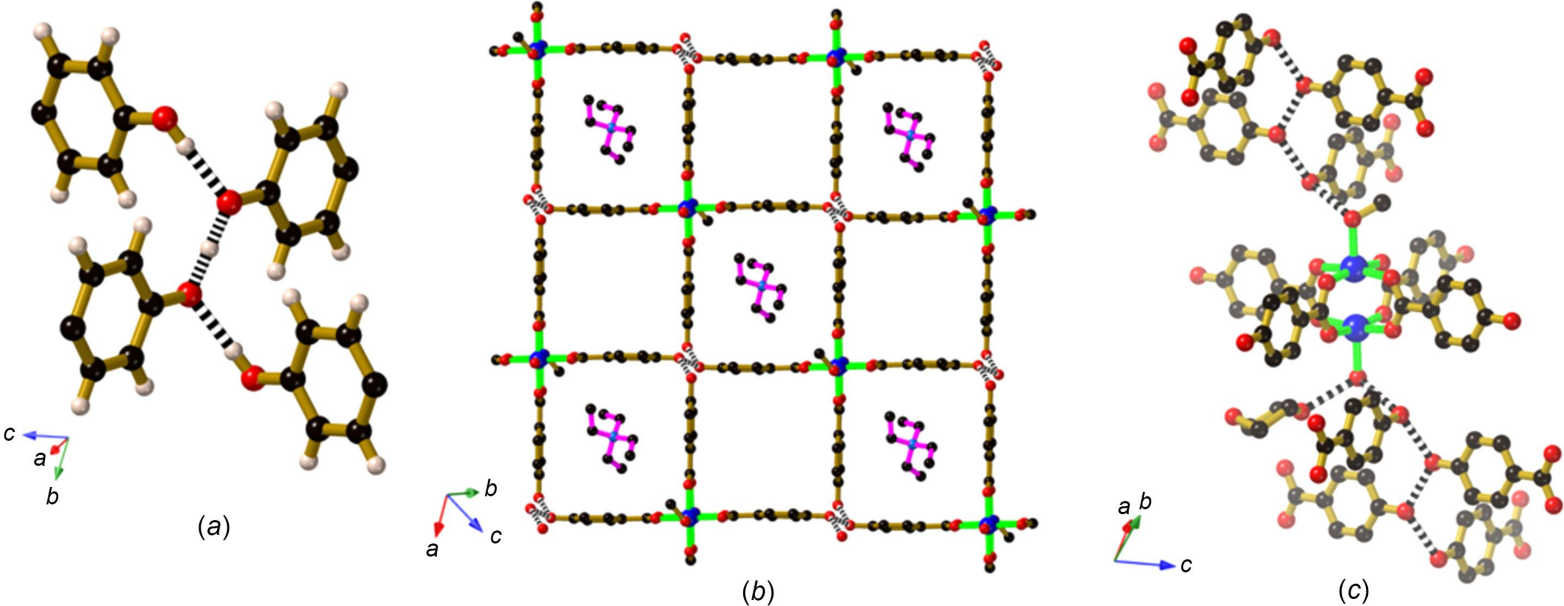
The structure of [NEt_4_][Cu_2_(Hhba)_2_(H_0.5_hba)_2_(CH_3_OH)(H_2_O)]·2(di­ox­ane) (**2**), showing (*a*) the hy­dro­gen-bonded 4-con­nected node, (*b*) the 4,4-network with the di­ox­ane mol­ecules and NEt_4_^+^ ions in the square cavities (only one of the two orientations of each tetra­ethyl­ammonium ion is shown), and (*c*) the inter­sheet hy­dro­gen-bond inter­actions involving the coordinated water/me­tha­nol mol­ecules which extend into sheets above and below.

The com­pound [NEt_4_][Cu_2_(Hhba)_2_(H_0.5_hba)_2_(CH_3_OH)(H_2_O)]·2(di­ox­ane) (**2**) is formed by the combination of Cu_2_(OAc)_4_(H_2_O)_2_ and H_2_hba in di­ox­ane, followed by the addition of NEt_4_OH in an aqueous me­tha­nol solution. It was anti­cipated that the addition of the base (NEt_4_OH) would serve to generate at least some hba^2−^ anions. The Cu dimeric complex present in **1** is also evident in **2**, however, with disordered water and me­tha­nol mol­ecules occupying the axial sites in place of the di­ox­ane mol­ecules. A centre of inversion lies between the two Cu^II^ centres. The complex has formally lost one phenolic H atom, resulting in the formation of the anionic complex [Cu_2_(Hhba)_2_(H_0.5_hba)_2_(CH_3_OH)(H_2_O)]^−^. The four organic groups extend outwards towards the vertices of an approximate square and thus the complex may be con­sidered as a 4-con­necting node. Hydrogen bonding be­tween phenolic and phenolate groups results in the generation of a second 4-con­necting node within a two-dimensional (2D) network. As indicated in Fig. 4 ▸(*a*), the second type of 4-con­necting node consists of three H atoms that bind four O atoms in a zigzag arrangement. The two central O atoms are separated by a H atom, which was refined to a position midway between the two symmetry-related O atoms. The O⋯H⋯O separation of 2.446 (5) Å is indicative of a strong inter­action, and the dimer unit formed by this inter­action may be represented as (H_0.5_hba)_2_^3−^. Inter­estingly, the O⋯O separation between O atoms is similar to that seen in (H_1.5_hba)_2_^−^ (Abrahams *et al.*, 2021 ▸). Phenolic groups above and below the central pair of O atoms form additional hy­dro­gen bonds which are a little longer [O—H⋯O = 2.601 (4) Å], resulting in a short hy­dro­gen-bonded planar zigzag chain (O—H⋯O⋯H⋯O⋯H—O).

An anionic 2D 4,4-network (square-grid network) is generated by linking the [Cu_2_(Hhba)_2_(H_0.5_hba)_2_(CH_3_OH)(H_2_O)]^−^ complexes through hy­dro­gen bonds involving the phenolic/phenolate groups [Fig. 4 ▸(*b*)]. Two types of square-shaped cavities are formed within the network, with orientationally disordered tetra­ethyl­ammonium counter-ions occupying the larger cavities. Disordered di­ox­ane mol­ecules, which were crystallographically modelled with partial occupancies, occupy the slightly smaller square cavities and space between networks.

The 2D square-grid layers described above form hy­dro­gen bonds with adjacent parallel layers through the coordinated water/me­tha­nol mol­ecules. The coordinated mol­ecules serve as hy­dro­gen-bond donors, bonding to a terminus of the hy­dro­gen-bonded O—H⋯O⋯H⋯O⋯H—O chain. In the case of the coordinated water mol­ecule, a hy­dro­gen bond extends to a di­ox­ane mol­ecule located in one of the small square cavities of an adjacent sheet, as indicated in Fig. 4 ▸(*c*).

**Figure 5:**
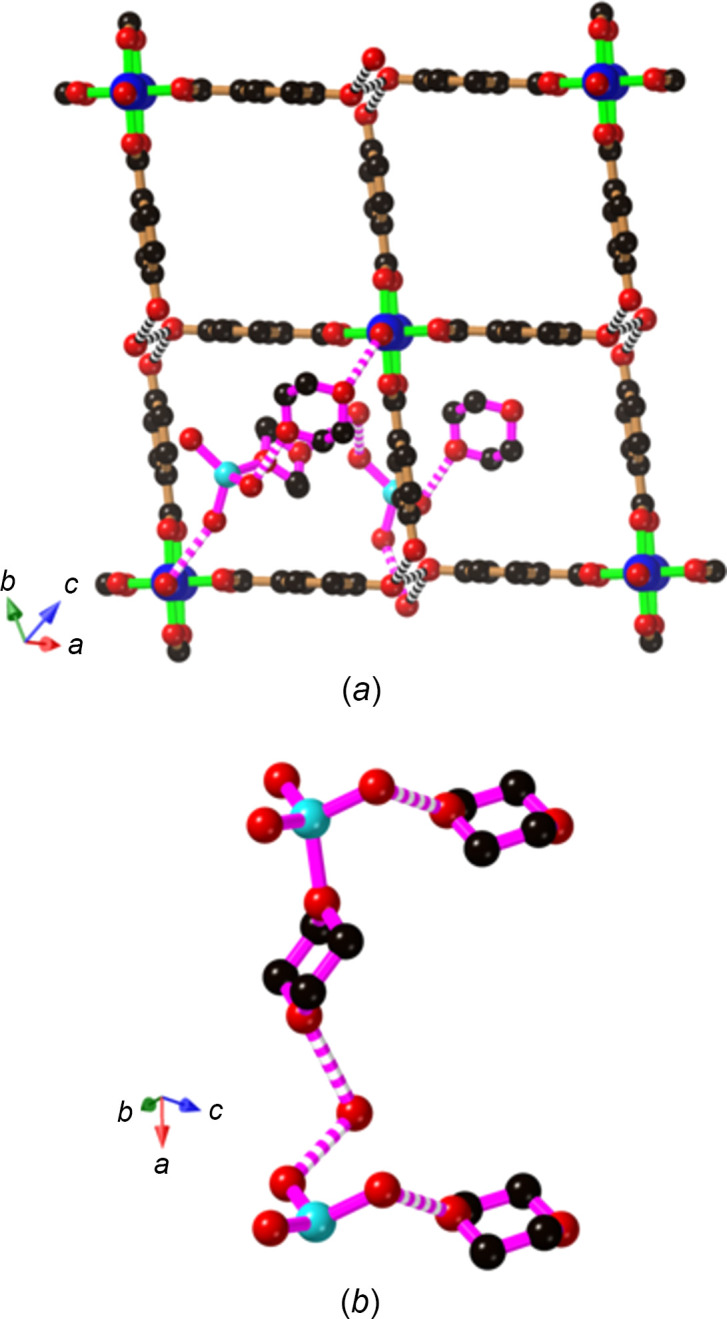
The structure of Li[Cu_2_(Hhba)_2_(H_0.5_hba)_2_(H_2_O)_2_]·3(di­ox­ane)·4H_2_O (**3**), showing (*a*) the square-grid structure with the Li^+^ cation coordinated by water and di­ox­ane mol­ecules located in the larger of the two types of square cavities, and (*b*) part of the infinite Li^+^–solvent chain.

Crystals of Li[Cu_2_(Hhba)_2_(H_0.5_hba)_2_(H_2_O)_2_]·3(di­ox­ane)·4H_2_O (**3**) are generated using LiOH as a base. A similar 4,4-network [Fig. 5 ▸(*a*)] to that found in **2** is formed. The binuclear unit is once again located on a centre of inversion and thus the asymmetric unit within the anionic network is very similar to that of com­pound **2**. As with **2**, there are two types of cavities in the sheet, one slightly larger than the other. The larger cavities, which are all crystallographically identical, are occupied by di­ox­ane mol­ecules, each of which is bound to a Li centre through one of the O atoms. Three water mol­ecules complete the coordination sphere of the Li centre. The other O atom of the di­ox­ane serves as a hy­dro­gen-bond acceptor to a water mol­ecule. A centre of inversion lies at the centre of the di­ox­ane and thus the Li^+^ centre is disordered across a pair of symmetry-related sites. These com­ponents are part of an infinite chain consisting of water mol­ecules, Li^+^ ions and bridging and branching di­ox­ane mol­ecules [Fig. 5 ▸(*b*)]. The chains pass through the larger square cavities of adjacent 4,4-networks, as indicated in Fig. 5 ▸(*a*). Branching di­ox­ane mol­ecules occupy the smaller square cavities.

**Figure 6:**
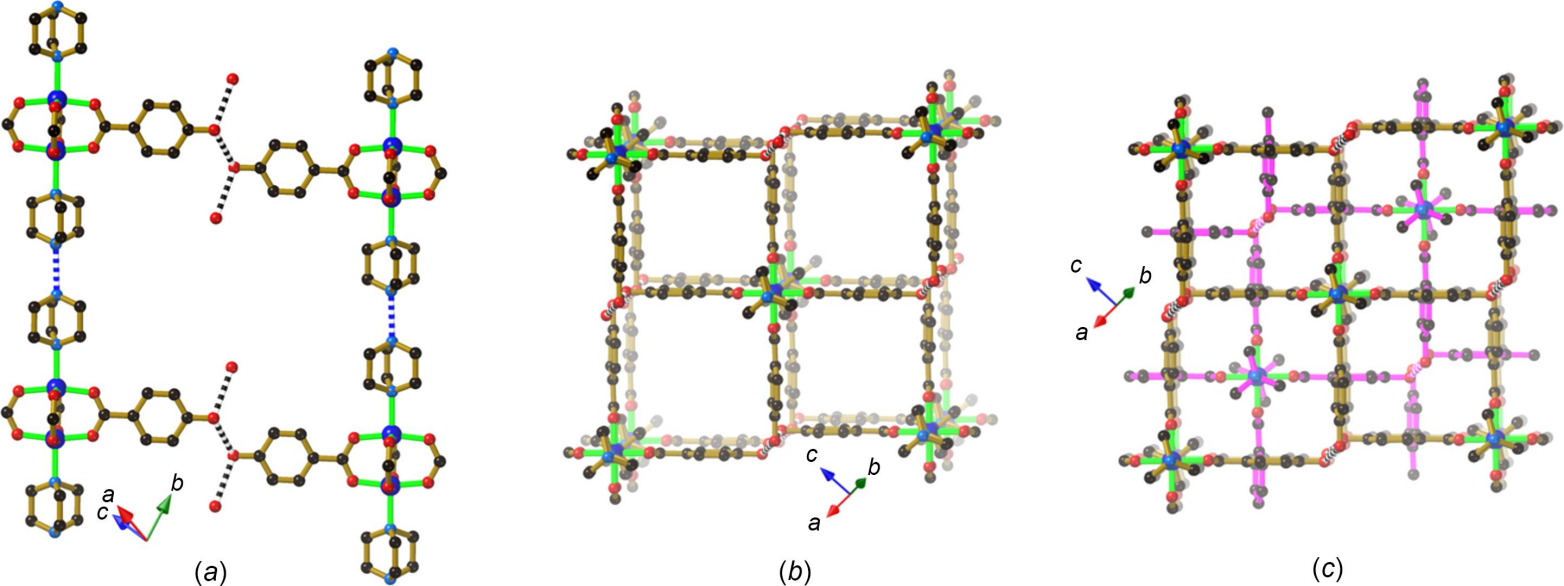
The structure of [Cu_2_(Hhba)_2_(H_0.5_hba)_2_(H_0.5_DABCO)_2_]·3CH_3_OH (**4**), showing (*a*) [DABCO⋯H⋯DABCO]^+^ links between [Cu_2_(Hhba)_2_(H_0.5_hba)_2_]^−^ hy­dro­gen-bonded sheets, (*b*) a single network with [Cu_2_(H_0.5_hba)_2_(Hhba)_2_]^−^ hy­dro­gen-bonded sheets linked by [DABCO⋯H⋯DABCO]^+^ con­nections and (*c*) a pair of inter­penetrating networks. Only a single orientation of each of the rotationally disordered DABCO units is represented in each figure.

The com­pound [Cu_2_(Hhba)_2_(H_0.5_hba)_2_(H_0.5_DABCO)_2_]·3CH_3_OH (**4**) is formed from the reaction of Cu_2_(OAc)_4_·2H_2_O and (H_0.5_DABCO)(H_1.5_hba) (**12**) in me­tha­nol. DABCO mol­ecules occupy the axial sites on the Cu dimer complex and are rotationally disordered around the axis which passes through the two N centres. A similar [Cu_2_(Hhba)_2_(H_0.5_hba)_2_]^−^ hy­dro­gen-bonded 2D network to that present in com­pounds **2** and **3** is apparent in **4**, but rather than cations occupying square cavities, the charge on the anionic complex is balanced by a proton located between a pair of coordinated DABCO ligands ([DABCO⋯H⋯DABCO]^+^), which forms a linear link between anionic sheets, as indicated in Fig. 6 ▸(*a*). The binuclear Cu complex now serves as a 6-con­necting node, with the DABCO ligands ex­ten­ding in a direction perpendicular to the familiar 2D network which was a feature of com­pounds **2** and **3** [Fig. 6 ▸(*b*)]. The result is a three-dimensional (3D) hy­dro­gen-bonded network with relatively large intra­framework voids that are large enough to accommodate a second equivalent network, as depicted in Fig. 6 ▸(*c*).

**Figure 7:**
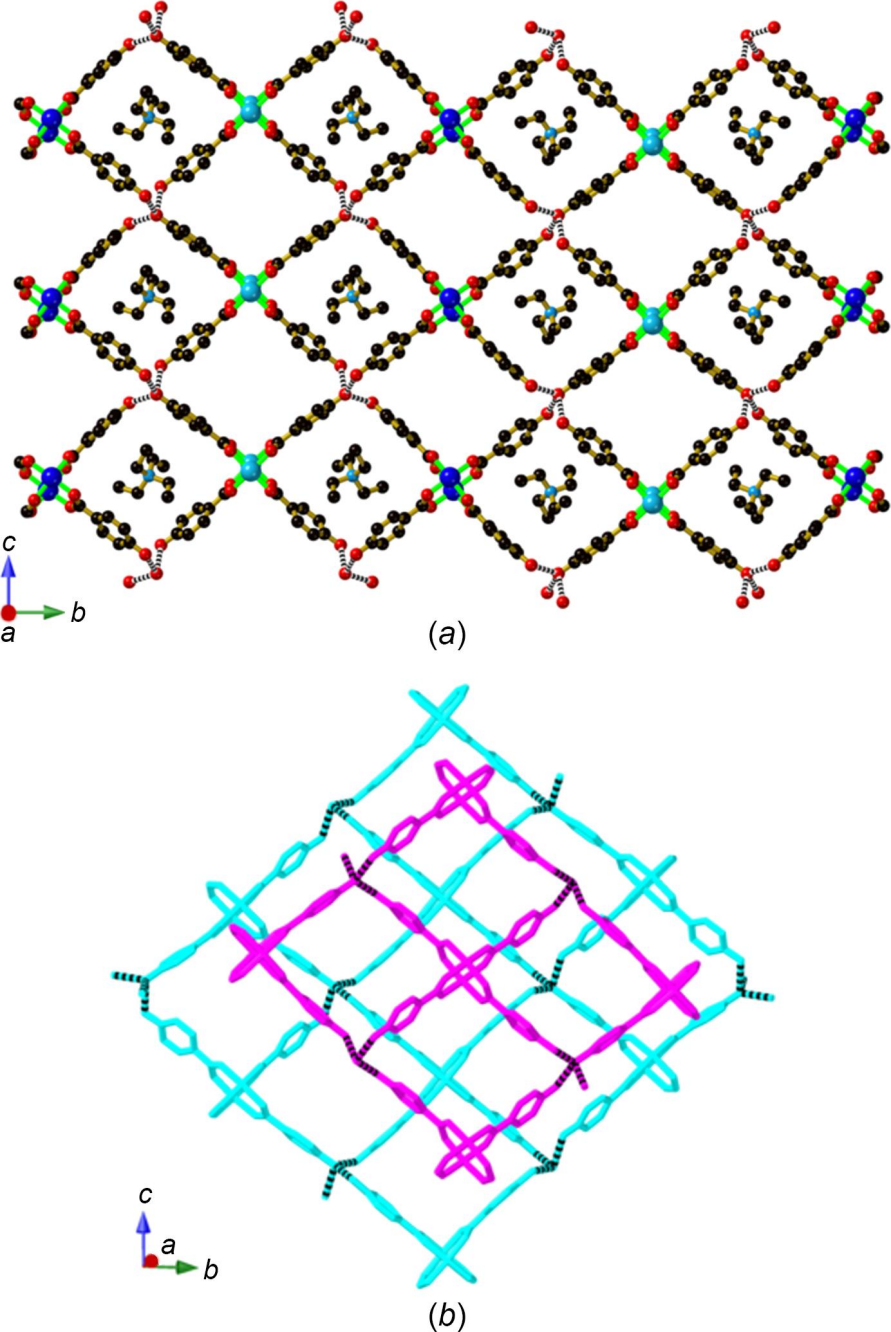
The structure of [Et_4_N]_2_[Cu_2_(Hhba)_2_(hba)_2_(di­ox­ane)_2_][Cu_2_(Hhba)_4_(di­ox­ane)(H_2_O)]·CH_3_OH (**5**), showing (*a*) the anionic 2D structure and (*b*) a pair of sheets indicating the *ABAB*… stacking.

An anionic 2D hy­dro­gen-bonded network is also a feature of the com­pound [Et_4_N]_2_[Cu_2_(Hhba)_2_(hba)_2_(di­ox­ane)_2_][Cu_2_(Hhba)_4_(di­ox­ane)(H_2_O)]·CH_3_OH (**5**), but there are clear dif­ferences in the 4-con­necting nodes that are formed by the aggregation of phenolic and phenolate groups. In each of the com­pounds **1**–**4**, there is only one crystallographically unique bi­nuclear complex, each possessing a centre of symmetry between the two Cu^II^ centres. In **5**, however, two crystallographically distinct binuclear Cu^II^ complexes are present. The first of these consists of four Hhba^−^ anions bound to a pair of centrosymmetrically-related Cu^II^ centres which are re­presented by dark-blue spheres in Fig. 7 ▸(*a*). The second com­plex is comprised of two crystallographically distinct Cu^II^ centres (the larger light-blue spheres) which are bound to two Hhba^−^ anions and two hba^2−^ anions. The hba^2−^ anions are *cis* to each other in the binuclear complex. The phenolate O atoms of the hba^2−^ anions serve as hy­dro­gen-bond acceptors from three phenolic groups, resulting in a 4-con­­necting hy­dro­gen-bonding node. The O⋯O separations in the [O^−^⋯(H—O)_3_] node are in the range 2.571 (2)–2.616 (2) Å. Charge balance for the anionic network is achieved with tetra­ethyl­ammonium ions that occupy every second square cavity of the 2D network, which extends in the *bc* plane. The 2D sheets stack in an *ABAB*… fashion [Fig. 7 ▸(*b*)], with tetra­ethyl­ammonium ions sandwiched be­tween [O^−^⋯(H—O)_3_] nodes of adjacent sheets. Dioxane and water mol­ecules, which are bound to the axial positions of the binuclear complexes, exhibit varying degrees of disorder and protrude into the vacant square cavities of adjacent sheets.

**Figure 8:**
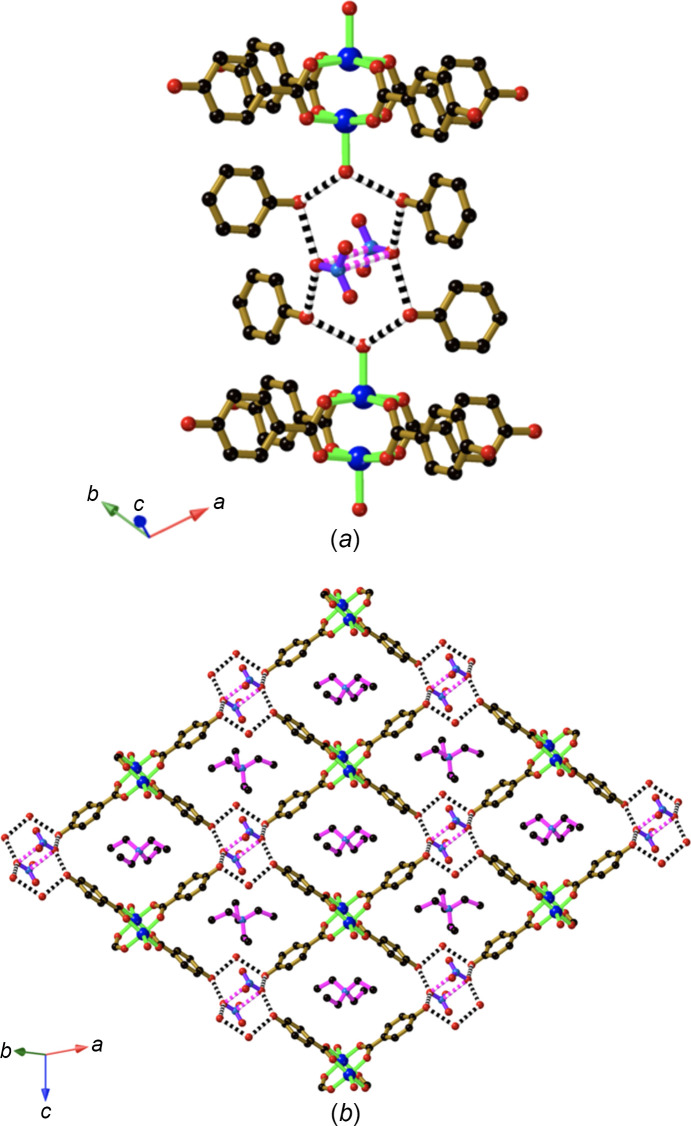
The structure of [Cu_2_(Hhba)_4_(H_2_O)_2_]·2(Et_4_NNO_3_) (**6**), showing (*a*) the hy­dro­gen bonding from four phenolic groups to a pair of nitrate anions, and from water mol­ecules to pairs of phenolic groups, and (*b*) the 2D network. Hydrogen bonds are indicated by black and white striped con­nections, whilst close inter­ionic contacts between nitrate anions are indicated by pink and white striped con­nections. Only a single orientation of each of the disordered Et_4_N^+^ units is represented in part (*b*).

The com­pound [Cu_2_(Hhba)_4_(H_2_O)_2_]·2Et_4_N(NO_3_) (**6**) is formed when Cu(NO_3_)_2_·3H_2_O is used as the source of Cu^II^ and the reaction mixture includes H_2_hba and Et_4_NOH. The expected binuclear complex, with water mol­ecules occupying the axial positions, is formed with a centre of symmetry between the metal centres. Each of the car­box­yl­ate ligands retains the phenolic proton to give the neutral [Cu_2_(Hhba)_4_(H_2_O)_2_] complex. Four phenolic groups combine with a pair of nitrate anions and a pair of coordinated water mol­ecules, from adjacent metal complexes, to form an unusual hy­dro­gen-bonded motif, as shown in Fig. 8 ▸(*a*). An inter­esting feature of this motif is the association of nitrate anion pairs, which make relatively close face-to-face contacts, with N⋯O separations of 2.883 (2) Å. Inspection of the Cambridge Structural Database (CSD, Version 5.45, March 2024 release; Groom *et al.*, 2016 ▸) reveals numerous examples of similar face-to-face inter­actions of nitrate anions. The aggregation of the phenolic groups, the two nitrate anions and a pair of coordinated water mol­ecules leads once again to the generation of a 2D network, as depicted in Fig. 8 ▸(*b*). If the link from the coordinated water mol­ecule to adjacent parallel layers is considered, then the overall structure is that of a 3D network. To balance the charge of the nitrate anion pairs, a tetra­ethyl­ammonium cation is required for every square cavity. There are two crystallographically distinct tetra­ethyl­ammonium ions, each of which is disordered over a pair of sites in each square cavity.

**Figure 9:**
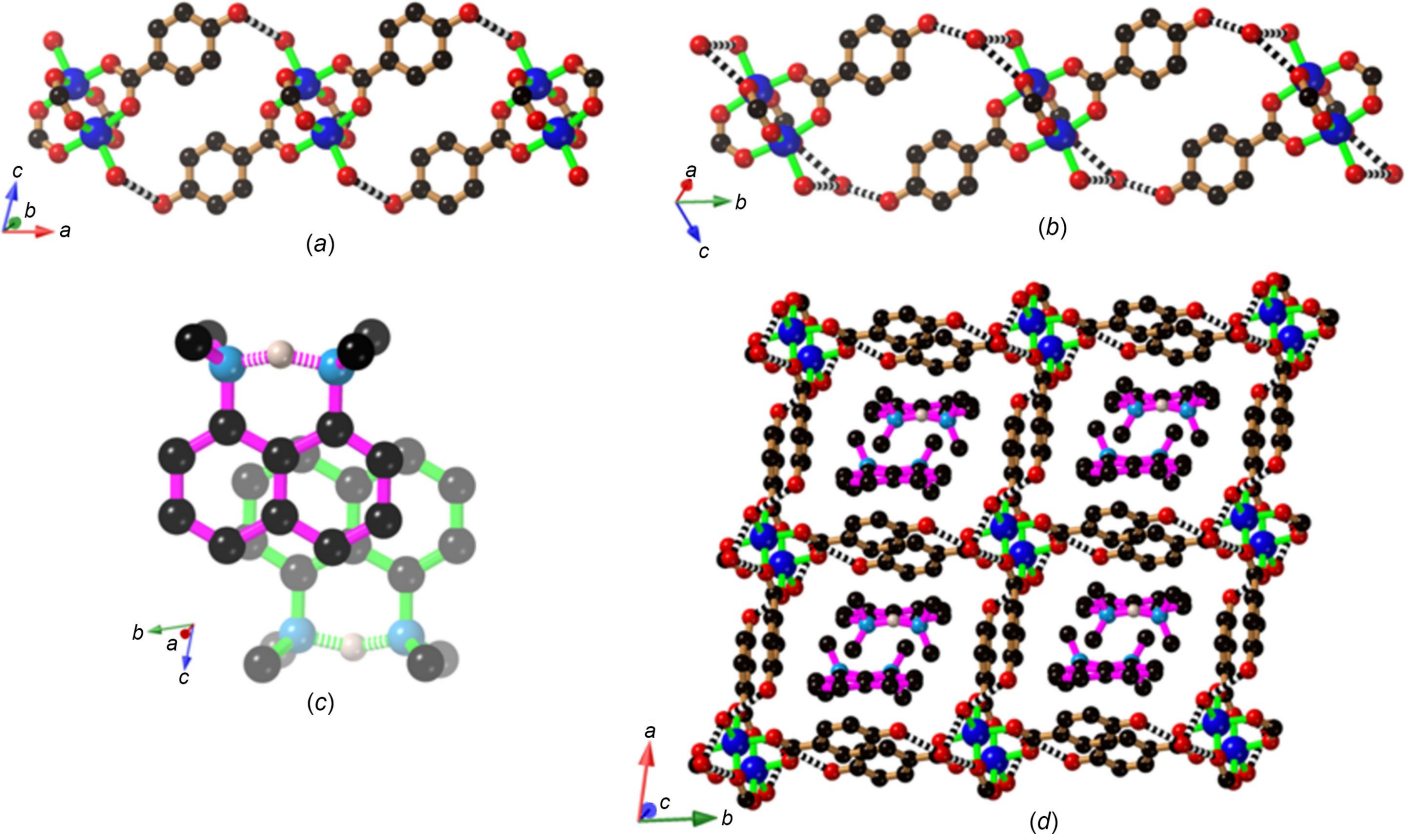
The structure of [Hbdn]_2_[Cu_2_(Hhba)_2_(hba)_2_(H_2_O)_2_]·3(di­ox­ane)·H_2_O (**7**), showing (*a*) the double hba^2−^ bridges between Cu^II^ pairs along the *a* axis, (*b*) the double Hhba^−^ bridges between Cu^II^ pairs along the *b* axis, with an additional water mol­ecule between the phenolic group and the coordinated water mol­ecule, (*c*) a pair of face-to-face Hbdn^+^ cations and (*d*) the 2D network ex­ten­ding in the *ab* plane, with the cations located in the cavities.

The employment of the organic base 1,8-bis­(di­methyl­amino)­naphthalene (bdn), more commonly known as a proton sponge, leads to the formation of crystals of com­position [Hbdn]_2_[Cu_2_(Hhba)_2_(hba)_2_(H_2_O)_2_]·3(di­ox­ane)·H_2_O (**7**). In this com­pound, the binuclear complex carries an overall 2− charge, with two hba^2−^ ligands *trans* to each other and ex­ten­ding along the *a*-axis direction. The *trans*-phenolate groups act as hy­dro­gen-bond acceptors from coordinated water mol­ecules belonging to complexes on either side along the direction of the *a* axis. In addition to acting as a proton acceptor *via* the *trans* hba^2−^ anions, the complex also serves as a proton donor to the complexes on either side through the coordinated water mol­ecules. Thus, each complex forms a pair of double hba^2−^ bridges, as indicated in Fig. 9 ▸(*a*). In addition to the centre of symmetry between the Cu^II^ centres, there is a centre of symmetry between the pair of hba^2−^ anions. Similarly, along the *b* axis, there are Hhba^−^ double bridges be­tween complexes with a non-coordinated water mol­ecule inter­spersed between the phenolic group and the coordinated water mol­ecule [Fig. 9 ▸(*b*)]. The presence of these two types of double bridges results in the generation of a 4,4-network in which the binuclear complex serves as the sole 4-con­necting node. Pairs of Hbdn^+^ cations, in a face-to-face arrangement, occupy the approximately square cavities [Figs. 9 ▸(*c*) and 9(*d*)]. The 2D layers that are generated are separated by disordered solvent mol­ecules.

**Figure 10:**
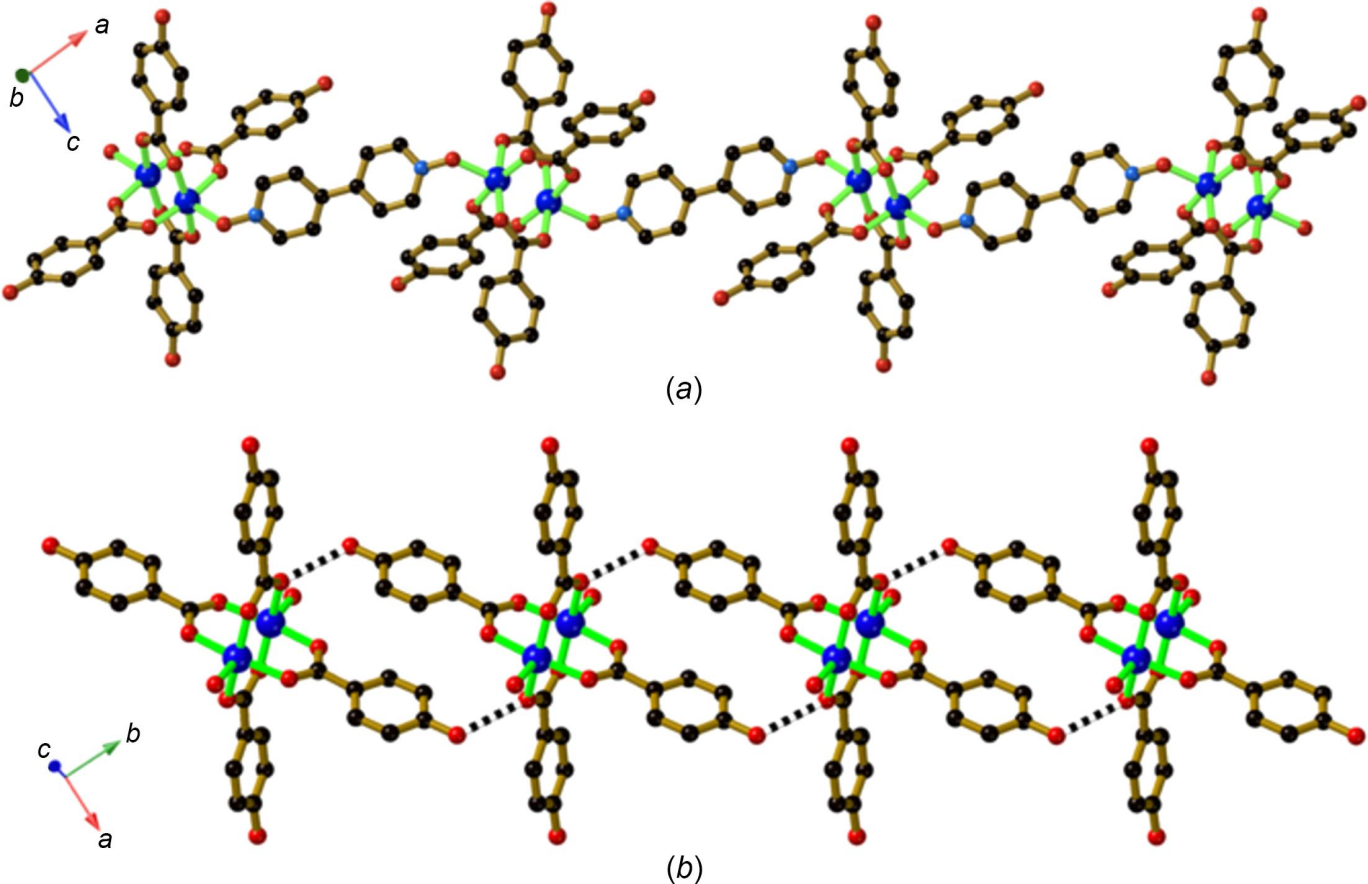
The structure of [Cu_2_(Hhba)_4_(O-bipy)]·H_2_O (**8**), showing (*a*) the 1D chains formed by the bridging O-bipy ligands and (*b*) the hy­dro­gen-bonded chains linking Cu_2_(Hhba)_4_ units together. The chains depicted in part (*b*) extend in both the [110] and the [1 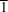 0] directions.

The addition of 4,4′-bi­pyridine *N*,*N*′-dioxide (O-bipy) to Cu(OAc)_2_·2H_2_O and H_2_hba under solvothermal conditions yields a one-dimensional (1D) coordination polymer of com­position [Cu_2_(Hhba)_4_(O-bipy)]·H_2_O (**8**). The O atoms of O-bipy coordinate to the axial positions of a binuclear Cu_2_(Hhba)_4_ complex, resulting in a coordination polymer chain that extends in the [201] direction [Fig. 10 ▸(*a*)]. Hydrogen-bonding inter­actions involving *trans*-Hhba^−^ anions lead to the generation of chains, as indicated in Fig. 10 ▸(*b*). Within the crystal structure, these hy­dro­gen-bonded chains extend in both the [110] and the [1 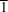 0] directions. If the links between Cu_2_(Hhba)_4_ units, shown in Figs. 10 ▸(*a*) and 10(*b*), are taken into account, the binuclear unit may be considered as a 4-con­nected node within a network which has the same topology as the CdSO_4_ net, *i.e.* Schlafli (point) symbol 6^5^·8^1^. Whilst the CdSO_4_ network is not as common as some of the better-known networks based on 4-con­necting nodes, there are examples re­por­ted in the literature including one based upon a Cu^II^–tetra­car­box­yl­ate dimer (Moulton *et al.*, 2003 ▸). The remaining two Hhba^−^ arms of the complex participate in hy­dro­gen-bonding inter­actions with non-coordinated water mol­ecules, which, in turn, form hy­dro­gen-bonded links to the coordinated O atoms of the O-bipy ligands. The result is a complex 3D hy­dro­gen-bonded network structure.

**Figure 11:**
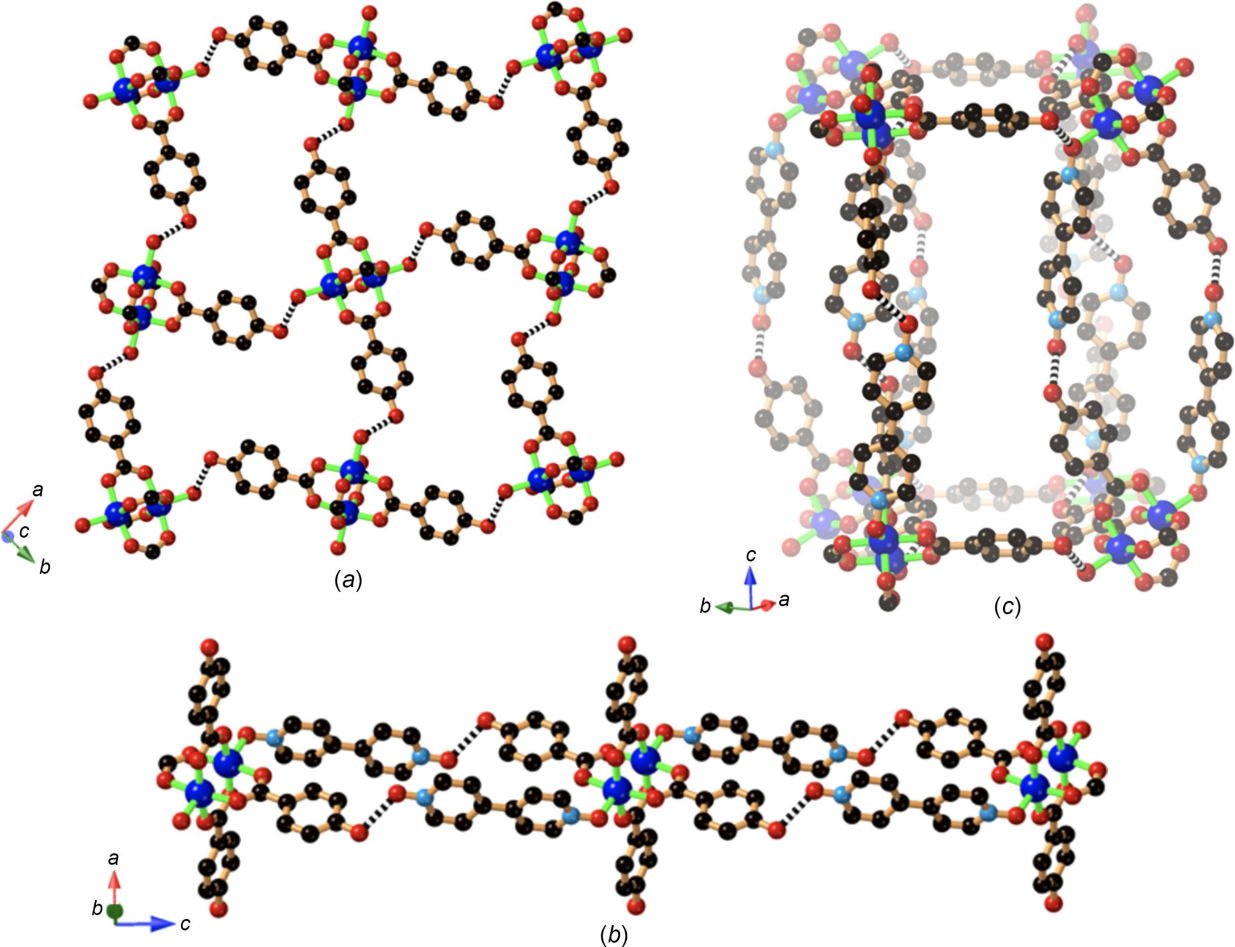
The structure of [Cu_2_(Hhba)_4_(O-bipy)_2_]·2(di­ox­ane) (**9**), showing (*a*) part of the hy­dro­gen-bonding 2D network that extends parallel to the *ab* plane, (*b*) double hy­dro­gen-bonded bridges that link the sheets depicted in part (*a*), and (*c*) part of the resulting 3D network.

Under solvothermal conditions using di­ox­ane as solvent, O-bipy reacts with Cu(OAc)_2_·2H_2_O and H_2_hba to form crystals of com­position [Cu_2_(Hhba)_4_(O-bipy)_2_]·2(di­ox­ane) (**9**), in which only one O atom of each O-bipy is coordinated to the neutral binuclear complex. Within this structure, a square-grid hy­dro­gen-bonded network lying parallel to the *ab* plane is generated, with links between phenolic groups and the coordinated O-bipy O atom, as indicated in Fig. 11 ▸(*a*). Additional con­nections from each binuclear unit extend along the *c* axis. These con­nections are double bridges and consist of a hy­dro­gen-bonding link between the non-coordinated O atom of O-bipy and a phenolic group of a Hhba^−^ ligand [Fig. 11 ▸(*b*)]. The double Hhba⋯O-bipy bridges ex­ten­ding from the bi­nuclear units to symmetry-related parallel units result in a 3D network that has the topology of the simple (primitive) cubic net, which is also known as the α-polonium net [Fig. 11 ▸(*c*)]. The intra­framework space is occupied by a symmetry-related second network.

**Figure 12:**
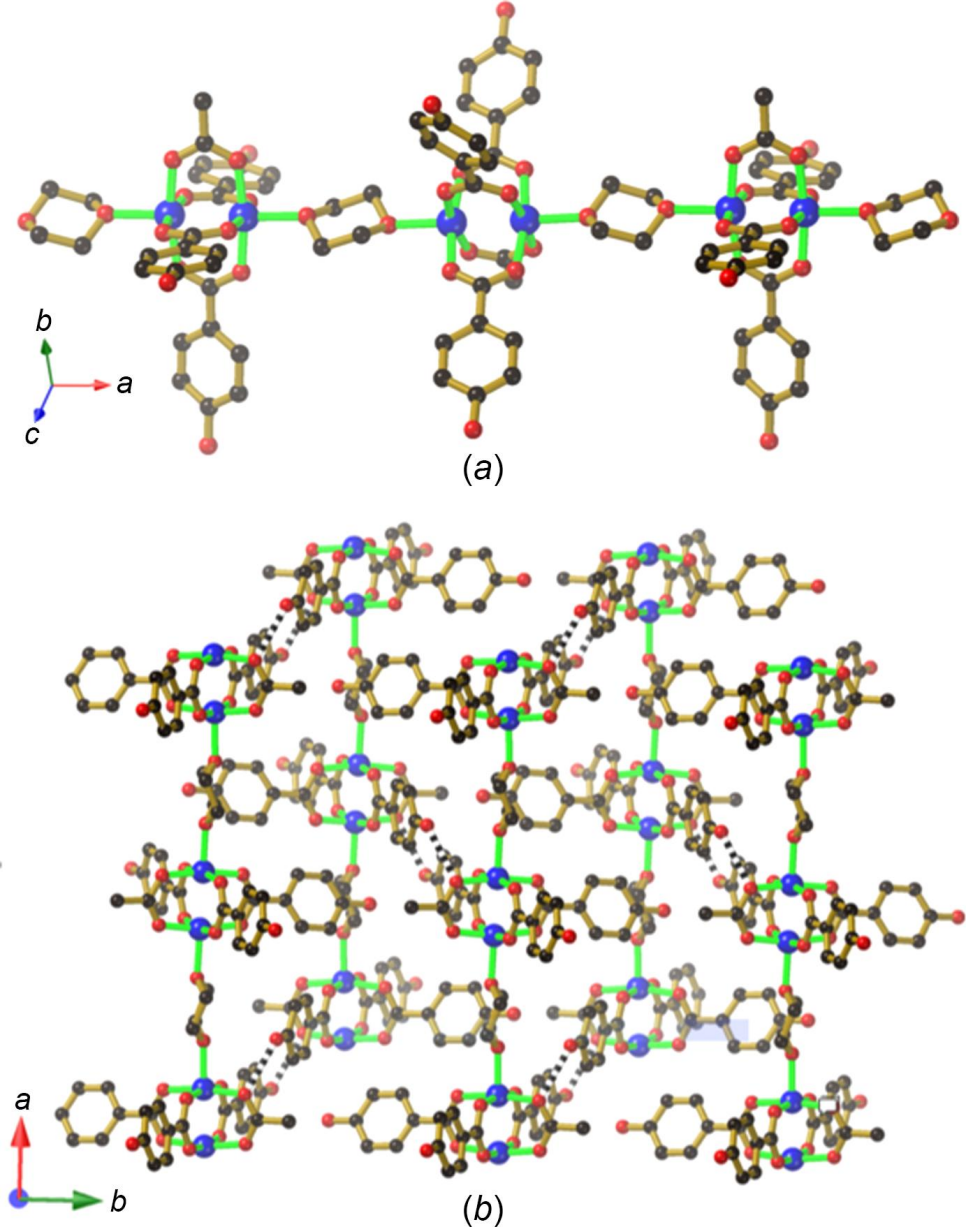
The linear chain structure of [Cu_2_(Hhba)_3_(OAc)(di­ox­ane)]·3.5(di­ox­ane) (**10**), showing (*a*) the chain formed by bridging di­ox­ane ligands that extends along the *a* axis and (*b*) the hy­dro­gen-bonded corrugated sheet network. Non-coordinated di­ox­ane mol­ecules have been omitted.

In the preparation of the copper com­pounds described above, except for **6**, the binuclear copper acetate complex served as the source of Cu^II^. In the following two examples (**10** and **11**), only partial replacement of the acetate by the Hhba^−^ ligands has occurred. In [Cu_2_(Hhba)_3_(OAc)(di­ox­ane)]·3.5(di­ox­ane) (**10**), the binuclear unit is again present, but with the Cu^II^ ions coordinated by three Hhba^−^ anions and one acetate anion. In this case, the binuclear units are bridged by di­ox­ane mol­ecules to form a chain that extends along the *a* axis, as indicated in Fig. 12 ▸(*a*). Hydrogen-bonding inter­actions involving the phenolic groups and acetate O atoms result in linked parallel chains [Fig. 12 ▸(*b*)]. The remaining phenolic groups inter­act with disordered di­ox­ane solvent mol­ecules.

**Figure 13:**
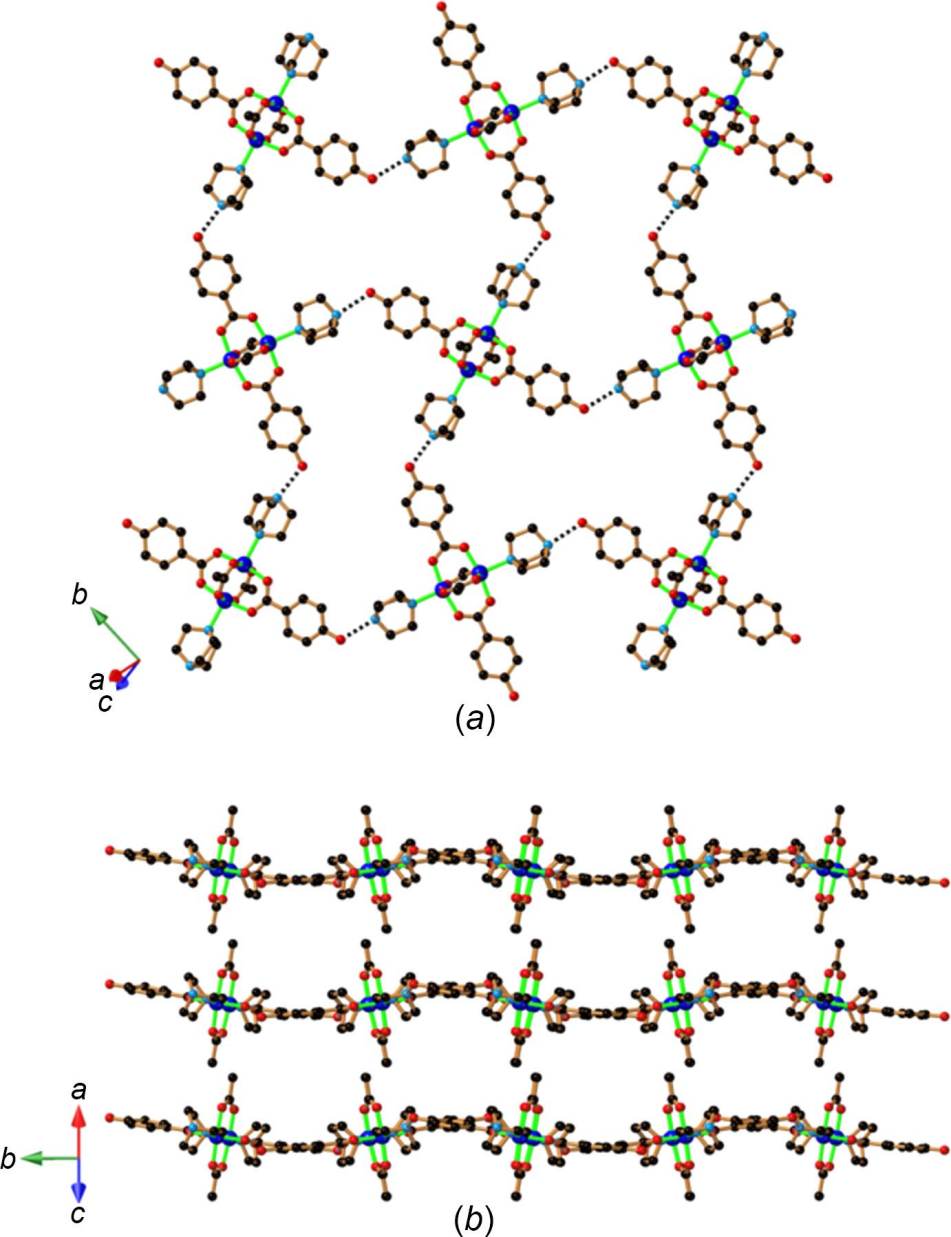
The structure of [Cu_2_(Hhba)_2_(OAc)_2_(DABCO)_2_]·10(di­ox­ane) (**11**), show­ing (*a*) the 2D hy­dro­gen-bonded network and (*b*) the crystal packing of the sheets.

In [Cu_2_(Hhba)_2_(OAc)_2_(DABCO)_2_]·10(di­ox­ane) (**11**), two *trans*-acetate anions remain coordinated, with coordinated DABCO mol­ecules occupying the axial positions in the complex. The [Cu_2_(Hhba)_2_(OAc)_2_(DABCO)_2_] unit serves as a 4-con­necting node within a 2D 4,4-network, as depicted in Fig. 13 ▸(*a*), with hy­dro­gen bonds formed between the phenolic groups and the non-coordinated DABCO N atom. Not sur­prisingly, there is a pronounced bend in the con­nection between the complexes associated with the C—O—H bond angle. The 2D layers stack on top of each other, with the methyl groups of the acetate ion ex­ten­ding above and below the mean plane of the 2D network [Fig. 13 ▸(*b*)]. Dioxane solvent mol­ecules occupy the voids between neighbouring sheets.

The final com­pound described in this article is the 1:1 cocrystal formed from the combination of DABCO and H_2_hba, which was used as a reactant in the formation of com­pound **4**.

**Figure 14:**
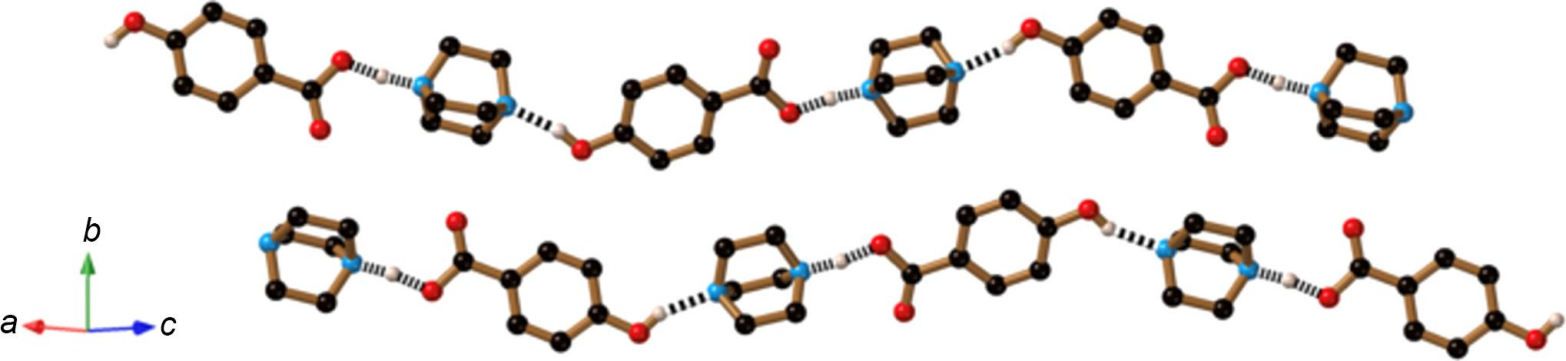
The crystal structure of (H_0.5_DABCO)(H_1.5_hba) (**12**), showing the formation of hydrogen-bonded chains that extend in the [10 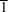 ] direction.

The crystal structure determination indicates two DABCO and two 4-hy­droxy­benzoic acid mol­ecules in the asymmetric unit. Hydrogen-bonded chains that extend in the [10 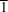 ] direction are generated as indicated in Fig. 14 ▸. Each chain contains only one type of DABCO and 4-hy­droxy­benzoic acid mol­ecule. The orientations of the 4-hy­droxy­benzoic acid in the two crystallographically distinct chains, as represented in Fig. 14 ▸, are opposite to each other and thus the two chains are anti­parallel. The cocrystal adopts the centrosymmetric space group *P*2_1_/*n* and thus each chain depicted in Fig. 14 ▸ also has a symmetry-related counterpart which is anti­parallel.

In the structure refinement, peaks of electron density near C atoms were consistent with the expected positions for H atoms. Similarly, electron-density peaks corresponding to the phenolic H atom appeared at a distance consistent with an O—H covalent bond. For each of the two carb­oxy­lic acid groups, peaks of electron density are located at positions further from the O atom than expected based upon normal O—H covalent bonds. These peaks were assigned as H atoms and the position of each was refined, leading to O⋯H separations of 1.28 (3) Å. The assigned H atoms are 1.25 (3) and 1.24 (3) Å from the N atoms of the neighbouring DABCO mol­ecules. The two N⋯O separations of 2.518 (2) and 2.515 (2) Å are remarkably short for N⋯O hy­dro­gen bonds. The N⋯O separations between the phenolic O atom and the neighbouring N atom of the DABCO are significantly longer [2.640 (2) and 2.619 (2) Å], but still relatively short for an N⋯O hy­dro­gen bond.

## General structural comments

For the Cu dimer com­pounds considered, com­pound **1** is the only one not to form a network-based material involving coordinate and/or hy­dro­gen-bond inter­actions. The failure of **1** to form a network may be attributed to the di­ox­ane mol­ecules which bind to the phenolic groups of the neutral Cu_2_(Hhba)_4_ units and block any prospect of network formation. In com­pounds **7**–**11**, the Cu dimer unit serves as a single node within a 2D (**7**, **10** and **11**) or 3D (**8** and **9**) network, whilst in **2**–**6**, there are two types of nodes.

In com­pounds **2**–**6**, the Cu dimer serves as a node, with a second node formed from either the aggregation of phenolic and phenolate groups (**2**–**5**) or, alternatively, from the involvement of nitrate anions (**6**). In the networks involving two types of nodes (**2**–**6**), cations are present in the structure. In the structures of **2**, **3**, **5** and **6**, the cations occupy square-shaped cavities, whereas in **4**, a proton shared between the non-coordinated N atoms of DABCO ligands serves as a link between Cu dimer nodes.

An average charge of 1− per Cu dimer unit appears to favour the formation of a second type of node in the hy­dro­gen-bonded networks that are generated. In each of **2**–**5**, four O atoms are held together by three H atoms. Whilst the hy­dro­gen-bonded nodes formed in **2**–**4** are similar, consisting of a planar zigzag arrangement of O atoms with a short hy­dro­gen bond between the central two O atoms, the corresponding node in **5** consists of a clearly defined phenolate O atom serving as a hy­dro­gen-bond acceptor from three phenol groups. In the remaining structures (**7**–**11**), the phenol groups only serve to provide links between Cu dimer nodes.

In com­pounds **8**–**11**, the Cu dimer unit is neutral, with all hba ligands present as Hhba^−^. These circumstances appear to favour single node (Cu dimer) networks with phenolic groups facilitating links between nodes rather than forming nodes based upon the aggregation of phenolic groups. Although four phenolic groups are involved in a second type of node in **6**, the nitrate ion pairs play a key role in the generation of the second type of node.

Compound **7** represents a special case in which the Cu dimer has a 2− charge, *i.e.* [Cu_2_(Hhba)_2_(hba)_2_]^2−^. This di­anion is also present in **5**, but the presence of equal numbers of neutral [Cu_2_(Hhba)_4_] means that the average charge on a Cu dimer in **5** is 1−. On the basis of the other structures, it might be expected that the presence of phenolic and phenolate groups in **7** would promote the formation of a second type of node, but instead, only one type of node is apparent with double ligand bridges between the Cu dimer nodes. A possible explanation for the adoption of the network, which is depicted in Fig. 9 ▸(*b*), is the complementary inter­action between pairs of face-to-face Hbdn^+^ cations and the anionic network con­taining appropriately sized cavities.

Compounds **10** and **11** represent rare examples of mixed car­box­yl­ate systems for binuclear copper(II) dimers. According to Hassanein *et al.* (2015 ▸), of the 1300 Cu car­box­yl­ate dimers re­por­ted, only nine were heteroleptic. In the specific case of acetate-containing dimers, the CSD contains just one com­pound (refcode HIQQEE02; Luo *et al.*, 2010 ▸) with one acetate ligand in combination with three other ferrocene­car­box­yl­ate ligands.

In the structures described in this investigation, there are examples of H atoms refining to a position halfway or nearly halfway between a pair of atoms which form a hy­dro­gen bond. The limitations of X-ray diffraction data in accurately determining the positions of H atoms make it difficult to distinguish between disordered asymmetric hy­dro­gen bonds and truly symmetric hy­dro­gen bonds. In the cases where we have refined the structure with a symmetric (or close to symmetric) hy­dro­gen bond, we wish to stress that the assignments of the H-atom positions are only tentative.

## Conclusion

The network structures described in this work demonstrate that Cu dimer units involving the hba^2−^/Hhba^−^ ligands can form a variety of networks. Clearly, hy­dro­gen bonding has played a major role in the formation of the networks. Of particular importance has been the network nodes formed from multiple hy­dro­gen bonds. In com­pounds **2**–**4**, the planar zigzag motif has shown itself to be an effective 4-con­necting node with the ligands ex­ten­ding outwards to the corners of an approximate square. A different type of hy­dro­gen-bonding motif involving three phenol groups serving as donors to a single phenolate group can also produce a 4-con­necting node, as seen in **5**.

An unusual and beautiful example of a hy­dro­gen-bonding node is apparent in **6**, where a pair of nitrate anions, four phenol groups and a pair of coordinated water mol­ecules combine to generate a network which accommodates tetra­ethyl­ammonium cations in square windows. In the remaining structures involving copper dimer complexes, hy­dro­gen bonding plays a key structural role in the arrangement of the mol­ecular com­ponents although they do not contain hy­dro­gen-bonded nodes within networks.

Clearly there are numerous opportunities for exploiting the ability of the phenolic/phenolate groups within Cu^II^ tetra­car­box­yl­ate dimers to create a wide variety of extended structures. Furthermore, the work has demonstrated, using co­lig­ands such as DABCO/HDABCO^+^ and O-bipy, that additional con­nectivity of the Cu dimer can be achieved by utilizing the axial positions on the Cu centres. The use of different countercations provides further opportunities for crystal engineering. For these reasons, and given the diversity of structures described in this current work, there would appear to be plenty of scope for ex­ten­ding this family of com­pounds.

## Supplementary Material

Crystal structure: contains datablock(s) compound_1, compound_2, compound_3, compound_4, compound_5, compound_6, compound_7, compound_8, compound_9, compound_10, compound_11, compound_12, global. DOI: 10.1107/S2053229624004534/jx3083sup1.cif

Structure factors: contains datablock(s) compound_1. DOI: 10.1107/S2053229624004534/jx3083compound_1sup2.hkl

Structure factors: contains datablock(s) compound_2. DOI: 10.1107/S2053229624004534/jx3083compound_2sup3.hkl

Structure factors: contains datablock(s) compound_3. DOI: 10.1107/S2053229624004534/jx3083compound_3sup4.hkl

Structure factors: contains datablock(s) compound_4. DOI: 10.1107/S2053229624004534/jx3083compound_4sup5.hkl

Structure factors: contains datablock(s) compound_5. DOI: 10.1107/S2053229624004534/jx3083compound_5sup6.hkl

Structure factors: contains datablock(s) compound_6. DOI: 10.1107/S2053229624004534/jx3083compound_6sup7.hkl

Structure factors: contains datablock(s) compound_7. DOI: 10.1107/S2053229624004534/jx3083compound_7sup8.hkl

Structure factors: contains datablock(s) compound_8. DOI: 10.1107/S2053229624004534/jx3083compound_8sup9.hkl

Structure factors: contains datablock(s) compound_9. DOI: 10.1107/S2053229624004534/jx3083compound_9sup10.hkl

Structure factors: contains datablock(s) compound_10. DOI: 10.1107/S2053229624004534/jx3083compound_10sup11.hkl

Structure factors: contains datablock(s) compound_11. DOI: 10.1107/S2053229624004534/jx3083compound_11sup12.hkl

Structure factors: contains datablock(s) compound_12. DOI: 10.1107/S2053229624004534/jx3083compound_12sup13.hkl

CCDC references: 2355537, 2355536, 2355535, 2355534, 2355533, 2355532, 2355531, 2355530, 2355529, 2355528, 2355527, 2355526

## Figures and Tables

**Figure 2:**
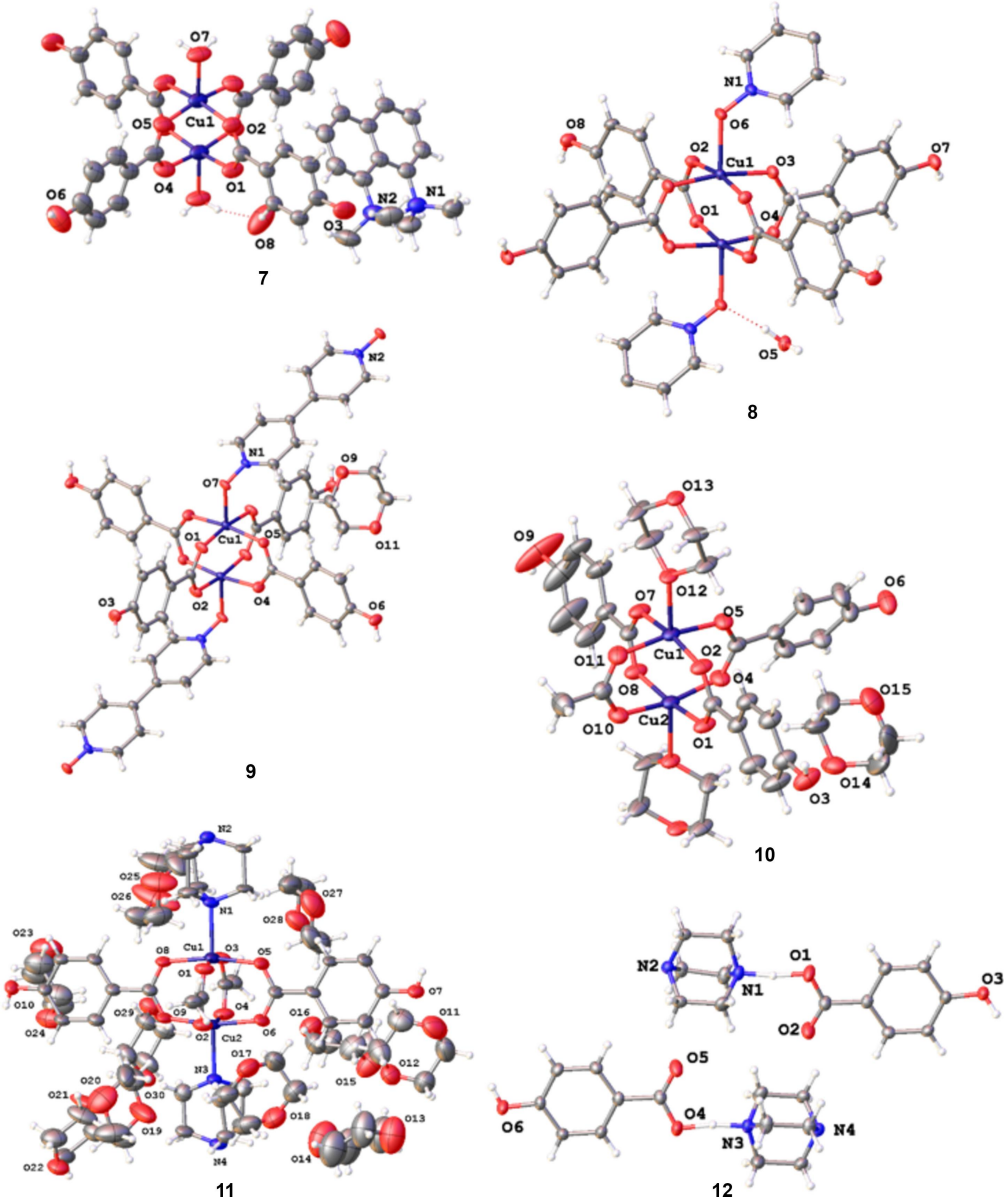
The structures of com­pounds **7**–**11**, showing the copper dimer units and the atom-labelling schemes. The structure of the cocrystal formed from the combination of H_2_hba and DABCO (**12**) is also shown. For clarity, the labels of the C and H atoms are not shown. Displacement ellipsoids are drawn at the 50% probability level. H atoms are represented by spheres of arbitrary size. The red dotted lines represent hy­dro­gen-bonding inter­actions. In **9**, only one configuration of the disordered di­ox­ane mol­ecules is shown, and in **11**, only one configuration of the DABCO mol­ecules is shown.

**Table:** Data for com­pounds **4** and **5** were collected at the Australian Synchrotron (beamlines MX1 and MX2, respectively). An Oxford Diffraction Supernova diffractometer was used for com­pounds **8** and **12**. Other diffraction data were measured using a Rigaku XtalLAB Synergy S (Dualflex, HyPix) diffractometer. Cu *K*α^1^ radiation was employed on the laboratory-based diffractometers. Absorption corrections on the synchrotron data employed the multi-scan method using *XDS* (Kabsch, 2010 ▸). The multi-scan method was also used for diffractometer data employing *CrysAlis PRO* (Rigaku OD, 2018 ▸). Data were collected at 100 K, except for **8** and **12** (130 K). H atoms were treated by a mixture of independent and constrained refinement.

	**1**	**2**	**3**	**4**
Crystal data				
Chemical formula	[Cu_2_(C_7_H_5_O_3_)_4_(C_4_H_8_O_2_)_2_]·4C_4_H_8_O_2_	(C_8_H_20_N)[Cu_2_(C_7_H_5_O_3_)_2_(C_7_H_4.5_O_3_)_2_(CH_4_O)(H_2_O)]·2C_4_H_8_O_2_	[Li(C_4_H_8_O_2_)(H_2_O)_3_][Cu_2_(C_7_H_5_O_3_)_2_(C_7_H_4.5_O_3_)_2_(H_2_O)_2_]·2C_4_H_8_O_2_·H_2_O	[Cu_2_(C_7_H_5_O_3_)_2_(C_7_H_4.5_O_3_)_2_(C_6_H_12.5_N_2_)_2_]·3CH_4_O
*M*_r_	1204.14	1031.02	1047.81	995.99
Crystal system, space group	Orthorhombic, *P* *c* *c* *n*	Triclinic, *P* 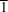	Triclinic, *P* 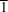	Triclinic, *P* 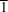
*a*, *b*, *c* (Å)	27.7701 (8), 21.2278 (4), 9.3888 (1)	10.1659 (4), 10.7383 (6), 12.9878 (4)	9.7711 (8), 10.1924 (6), 12.5863 (7)	8.8860 (18), 10.702 (2), 12.360 (3)
α, β, γ (°)	90, 90, 90	76.132 (4), 69.646 (3), 75.304 (4)	73.615 (5), 79.528 (6), 83.412 (6)	73.13 (3), 73.07 (3), 81.68 (3)
*V* (Å^3^)	5534.7 (2)	1267.74 (10)	1179.86 (15)	1073.8 (4)
*Z*	4	1	1	1
μ (mm^−1^)	1.66	1.63	1.85	1.07
Crystal size (mm)	0.14 × 0.13 × 0.05	0.17 × 0.12 × 0.05	0.14 × 0.07 × 0.03	0.18 × 0.11 × 0.07

Data collection				
*T*_min_, *T*_max_	0.567, 1.000	0.885, 1.000	0.610, 1.000	0.321, 0.432
No. of measured, independent and observed [*I* > 2σ(*I*)] reflections	18728, 5586, 4112	17988, 5262, 4672	10093, 4145, 3048	15021, 3849, 2994
*R*_int_	0.049	0.047	0.070	0.061
(sin θ/λ)_max_ (Å^−1^)	0.634	0.634	0.602	0.617

Refinement				
*R*[*F* ^2^ > 2σ(*F* ^2^)], *wR*(*F* ^2^), *S*	0.063, 0.187, 1.04	0.068, 0.200, 1.06	0.089, 0.261, 1.09	0.089, 0.263, 1.05
No. of reflections	5586	5262	4145	3849
No. of parameters	354	459	338	325
No. of restraints	2	419	42	364
Δρ_max_, Δρ_min_ (e Å^−3^)	0.47, −0.48	0.73, −1.00	1.21, −1.32	1.14, −0.76

**Table:** 

	**5**	**6**	**7**	**8**
Crystal data				
Chemical formula	(C_8_H_20_N)[Cu_2_(C_7_H_4_O_3_)_2_(C_7_H_5_O_3_)_2_(C_4_H_8_O_2_)_2_][Cu_2_(C_7_H_5_O_3_)_4_(C_4_H_8_O_2_)(H_2_O)]·CH_4_O	(C_8_H_20_N)_2_[Cu_2_(C_7_H_5_O_3_)_4_(H_2_O)_2_](NO_3_)_2_	(C_14_H_19_N_2_)_2_[Cu_2_(C_7_H_5_O_3_)_2_(C_7_H_4_O_3_)_2_(H_2_O)_2_]·3(C_4_H_8_O_2_)·H_2_O	[Cu_2_(C_7_H_5_O_3_)_4_(C_10_H_8_N_2_O_2_)]·H_2_O
*M*_r_	1923.89	1096.07	1420.47	881.72
Crystal system, space group	Orthorhombic, *P* *n* *m* *a*	Triclinic, *P* 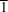	Triclinic, *P* 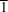	Monoclinic, *C*2/*c*
*a*, *b*, *c* (Å)	12.833 (3), 54.548 (11), 12.119 (2)	10.4964 (3), 10.6595 (2), 12.3226 (3)	10.2155 (2), 12.0897 (3), 15.7111 (4)	11.9851 (8), 17.7472 (11), 17.1294 (10)
α, β, γ (°)	90, 90, 90	97.271 (2), 101.042 (2), 108.130 (2)	69.581 (3), 75.988 (2), 79.651 (2)	90, 92.637 (5), 90
*V* (Å^3^)	8483 (3)	1260.15 (6)	1754.64 (8)	3639.6 (4)
*Z*	4	1	1	4
μ (mm^−1^)	1.08	1.72	1.38	2.12
Crystal size (mm)	0.13 × 0.09 × 0.06	0.16 × 0.13 × 0.09	0.19 × 0.16 × 0.12	0.32 × 0.06 × 0.05

Data collection				
*T*_min_, *T*_max_	0.321, 0.432	0.720, 1.000	0.890, 1.000	0.898, 1.000
No. of measured, independent and observed [*I* > 2σ(*I*)] reflections	151309, 13753, 11493	14549, 5136, 4648	21601, 6618, 5772	6868, 3663, 3386
*R*_int_	0.054	0.048	0.036	0.024
(sin θ/λ)_max_ (Å^−1^)	0.753	0.634	0.610	0.629

Refinement				
*R*[*F* ^2^ > 2σ(*F* ^2^)], *wR*(*F* ^2^), *S*	0.050, 0.148, 1.02	0.038, 0.101, 1.08	0.064, 0.189, 1.05	0.034, 0.098, 1.05
No. of reflections	13753	5136	6618	3663
No. of parameters	585	408	361	263
No. of restraints	67	263	3	2
Δρ_max_, Δρ_min_ (e Å^−3^)	1.42, −0.84	0.47, −0.54	1.05, −0.62	0.36, −0.57

**Table:** 

	**9**	**10**	**11**	**12**
Crystal data				
Chemical formula	[Cu_2_(C_7_H_5_O_3_)_4_(C_10_H_8_N_2_O_2_)_2_]·2C_4_H_8_O_2_	[Cu_2_(C_7_H_5_O_3_)_3_(C_2_H_3_O_2_)(C_4_H_8_O_2_)]·3.5C_4_H_8_O_2_	[Cu_2_(C_7_H_5_O_3_)_2_(C_2_H_3_O_2_)_2_(C_6_H_12_N_2_)_2_]·10C_4_H_8_O_2_	C_6_H_13_N_2_^+^·C_7_H_5_O_3_^−^
*M*_r_	1228.09	993.92	1624.77	250.29
Crystal system, space group	Orthorhombic, *P* *b* *c* *a*	Monoclinic, *I*2/*a*	Monoclinic, *P* *c*	Monoclinic, *P*2_1_/*n*
*a*, *b*, *c* (Å)	17.5993 (3), 16.4583 (2), 19.7389 (2)	19.4801 (2), 13.0774 (2), 34.5443 (5)	9.7404 (3), 20.3001 (4), 22.9088 (7)	15.3994 (5), 10.6592 (3), 16.9261 (6)
α, β, γ (°)	90, 90, 90	90, 97.702 (1), 90	90, 118.494 (4), 90	90, 110.424 (4), 90
*V* (Å^3^)	5717.46 (13)	8720.7 (2)	3981.1 (2)	2603.67 (15)
*Z*	4	8	2	8
μ (mm^−1^)	1.59	1.90	1.36	0.75
Crystal size (mm)	0.09 × 0.07 × 0.05	0.26 × 0.09 × 0.06	0.34 × 0.25 × 0.14	0.22 × 0.16 × 0.13

Data collection				
*T*_min_, *T*_max_	0.344, 1.000	0.764, 1.000	0.533, 1.000	0.952, 1.000
No. of measured, independent and observed [*I* > 2σ(*I*)] reflections	23256, 5876, 4731	37963, 9032, 6906	27595, 12037, 9752	11079, 5386, 3805
*R*_int_	0.056	0.071	0.040	0.039
(sin θ/λ)_max_ (Å^−1^)	0.634	0.635	0.635	0.632

Refinement				
*R*[*F* ^2^ > 2σ(*F* ^2^)], *wR*(*F* ^2^), *S*	0.044, 0.129, 1.06	0.065, 0.191, 1.09	0.098, 0.287, 1.21	0.044, 0.120, 1.01
No. of reflections	5876	9032	12037	5386
Δρ_max_, Δρ_min_ (e Å^−3^)	0.59, −0.68	1.12, −1.24	1.38, −0.70	0.19, −0.24
Absolute structure	–	–	Refined as an inversion twin	–
Absolute structure parameter	–	–	0.46 (6)	–

Computer programs: *CrysAlis PRO* (Rigaku OD, 2015 ▸, 2018 ▸, 2021 ▸, 2023 ▸), *XDS* (Kabsch, 2010 ▸), *SHELXT* (Sheldrick, 2015*a* ▸), *olex2.solve* (Bourhis *et al.*, 2015 ▸), *SHELXL2018* (Sheldrick, 2015*b* ▸) and *OLEX2* (Dolomanov *et al.*, 2009 ▸).
